# Runt related transcription factor-1 plays a central role in vessel co-option of colorectal cancer liver metastases

**DOI:** 10.1038/s42003-021-02481-8

**Published:** 2021-08-10

**Authors:** Miran Rada, Audrey Kapelanski-Lamoureux, Stephanie Petrillo, Sébastien Tabariès, Peter Siegel, Andrew R. Reynolds, Anthoula Lazaris, Peter Metrakos

**Affiliations:** 1 Cancer Research Program, McGill University Health Centre Research Institute, Montreal, QC Canada; 2 Rosalind and Morris Goodman Cancer Research Centre, McGill University, Montreal, QC Canada; 3 Oncology R&D, AstraZeneca, Cambridge, United Kingdom

**Keywords:** Metastasis, Molecular biology, Tumour angiogenesis, Colorectal cancer

## Abstract

Colorectal cancer liver metastasis (CRCLM) has two major histopathological growth patterns: angiogenic desmoplastic and non-angiogenic replacement. The replacement lesions obtain their blood supply through vessel co-option, wherein the cancer cells hijack pre-existing blood vessels of the surrounding liver tissue. Consequentially, anti-angiogenic therapies are less efficacious in CRCLM patients with replacement lesions. However, the mechanisms which drive vessel co-option in the replacement lesions are unknown. Here, we show that Runt Related Transcription Factor-1 (RUNX1) overexpression in the cancer cells of the replacement lesions drives cancer cell motility via ARP2/3 to achieve vessel co-option. Furthermore, overexpression of RUNX1 in the cancer cells is mediated by Transforming Growth Factor Beta-1 (TGFβ1) and thrombospondin 1 (TSP1). Importantly, RUNX1 knockdown impaired the metastatic capability of colorectal cancer cells in vivo and induced the development of angiogenic lesions in liver. Our results confirm that RUNX1 may be a potential target to overcome vessel co-option in CRCLM.

## Abstract (web summary)

Miran Rada et al. identify a key role for RUNX1 in liver metastasis of colorectal cancer. They show that RUNX1 is necessary for driving blood vessel co-option from surrounding liver tissue and that regulation of this process relies on a signaling feedback loop between RUNX1 transcriptional target TSP1 in tumor cells and TGFβ1 in the liver.

## Introduction

Colorectal cancer (CRC) is the third most commonly diagnosed cancer in the world and ranking the second leading cause of cancer-related deaths in developed countries^1^. Since the majority of the intestinal mesenteric drainage enters the hepatic portal venous system, CRC metastases often spread to the liver, which is a major cause of mortality^2,3^. Currently, ablation with surgical resection is considered the only curative option resulting in 5-year survival rates of up to 50%. However, surgical treatment is not possible in 80% of colorectal cancer liver metastasis (CRCLM) cases and the patients are left with palliative options, mainly consisting of systemic treatment with palliative intent^2,4^.

Angiogenesis has been reported as an essential step in the growth of metastatic tumors, which is driven by vascular endothelial growth factor-A (VEGF-A)^5,6^. Therefore, numerous anti-angiogenic agents targeting VEGF signaling (e.g., Bevacizumab, Regorafenib, Aflibercept) combined with chemotherapy have been identified as an approved treatment in CRCLM patients to extend progression-free and/or overall survival^7–9^. However, a limited therapeutic benefit to overall survival (OS) has been observed^10^. The mechanisms that limit the therapeutic efficacy of anti-angiogenic therapy in patients are still unclear. One of the potential mechanisms that could explain resistance to anti-angiogenesis therapy is vessel co-option, a mechanism by which tumors obtain a blood supply without angiogenesis by exploiting pre-existing vasculature^9,11–13^. Since the tumor is co-opting the liver’s pre-existing vessels, anti-angiogenic treatment may have only a limited effect in vessel co-opting tumors. Vessel co-option has been reported in different cancers, for instance, liver metastases^14^, non–small cell lung cancer^15^, lung metastases^16,17^, lymph node metastasis^18,19^ and hepatocellular carcinoma^20^. Recent studies suggested two major distinct histopathological growth patterns (HGPs) of CRCLM lesions including desmoplastic HGP (DHGP) and replacement HGP (RHGP)^21,22^. DHGP lesions are characterized by a desmoplastic stromal layer that physically separates the cancer cells from the normal liver parenchyma. The cancer cells in DHGP lesions obtain their blood supply through angiogenesis^14,21,23^. However, the cancer cells in RHGP lesions infiltrate the liver parenchyma and replace the hepatocytes near the tumor periphery to co-opt pre-existing liver sinusoidal vessels instead of promoting angiogenesis^14,21,24^. Indeed, poor responses to anti-angiogenic therapy combined with chemotherapy have been reported in CRCLM patients with RHGP lesions compared to patients with DHGP lesions who achieve a better response to the same therapy^11,12,14,24,25^. It is worth mentioning that similar results were observed in breast^26^, uveal melanoma^27^, and pancreatic^28^ liver metastases.

Accumulating evidence suggests that cancer cell motility plays a pivotal role in the process of vessel co-option in various cancers^9,14,20,29^. Accordingly, Kuczynski et al.^20^ demonstrated upregulation of pathways involved in cancer cell motility and invasion in vessel co-opting hepatocellular carcinomas, such as epithelial-mesenchymal transition (EMT), STAT3 and Wnt/β-catenin signaling. The Actin-related protein 2/3 complex (ARP2/3) complex has also been implicated in tumor invasion, and a study from our group showed a positive correlation between ARP2/3 overexpression in cancer cells and CRCLM vessel co-option, and that ARP2/3 knockdown resulted in the conversion of CRCLM lesions with a vessel co-option phenotype to angiogenic lesions in vivo^14^. However, the molecular mechanisms by which the expression of ARP2/3 is upregulated in cancer cells are poorly understood.

The aim of this study was to understand the molecular mechanisms of vessel co-option in CRCLM. Herein, we identified RUNX1 as a key player in vessel co-option by regulating motility and EMT in cancer cells.

## Results

### RUNX1 is overexpressed in vessel co-opting CRCLM tumors

Our team previously reported ARP2/3 as a key mediator of vessel co-option in CRCLM^14^. The ARP2/3 complex is a stable multiprotein complex composed of seven subunits including ARP2 (ACTR2), ARP3, ARPC1 (p41), ARPC2 (p31), ARPC3 (p21), ARPC4 (p20), and ARPC5 (p16)^30^. Runt Related Transcription Factor-1 (RUNX1) is among the transcriptional factors that control the expression levels of ARP2/3^31–33^. It regulates the expression of various ARP2/3 subunits, such as ARPC1, ARPC2, and ARPC3^34^. RUNX1 is a member of the RUNX transcription factor family^35^. An abnormal elevation of RUNX1 has been reported in various cancers, for instance, breast cancer, colorectal cancer, pancreatic cancer, and brain cancer^36–38^. RUNX1 occupied thousands of genomic regions that corresponded to genes involved in tumor progression and angiogenesis^37,38^.

**Fig. 1:**
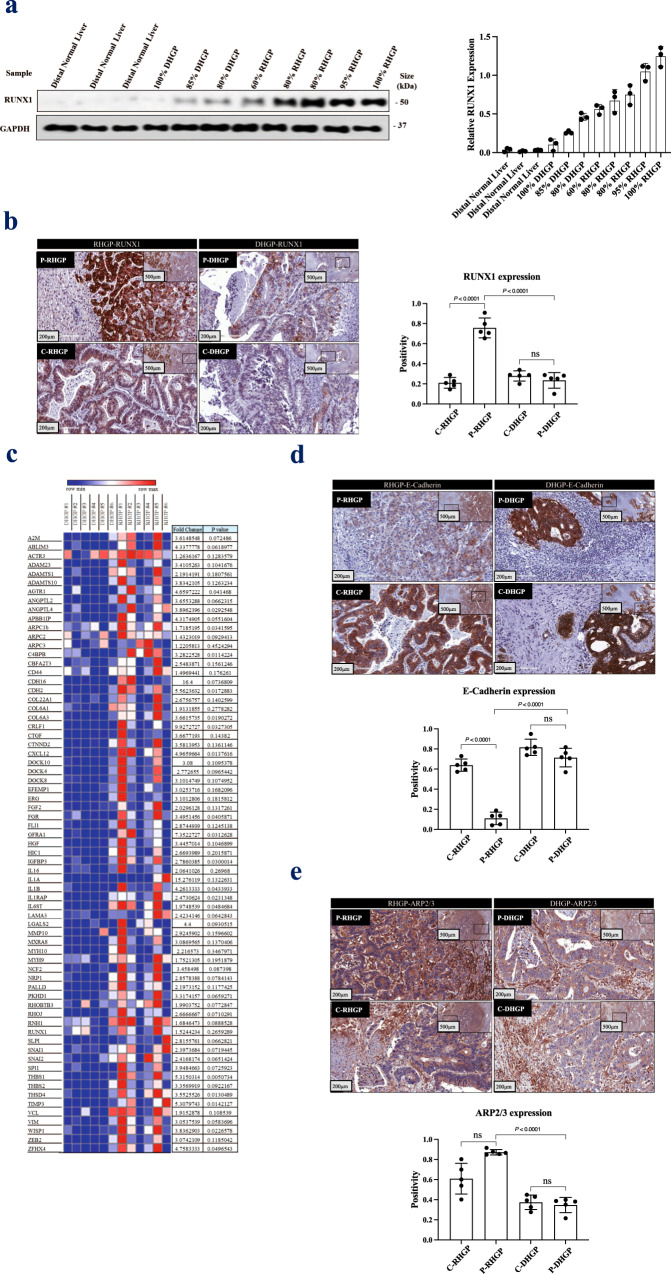
RUNX1 overexpressed in the replacement colorectal cancer liver metastasis lesions. **a** Western blot of RUNX1 in distal normal liver (control) and chemonaïve CRCLM cancer cells (left panel). The right panel represents the intensity of the bands (*n* = 3) that were determined using ImageJ software. **b** Immunohistochemistry staining of chemonaïve CRCLM lesions using an anti-RUNX1 antibody (left panel). Right panels show the positivity [total number of positive pixels/total number of pixels] that measured in RHGP (*n* = 5) and DHGP (*n* = 5) specimens using an optimized Aperio algorithm (mean + SD). C-RHGP Central tumor cells in RHGP lesions, P-RHGP Peripheral tumor cells in RHGP lesions, C-DHGP Central tumor cells in DHGP, P-DHGP Peripheral tumor cells in DHGP lesions. **c** Heatmap showing the expression of RUNX1 target genes in DHGP (*n* = 6) and RHGP (*n* = 6) CRCLM tumors. Red representing the highest levels of expression and blue representing the lowest levels of expression. **d**, **e** Immunohistochemistry staining of chemonaïve CRCLM lesions with E-cadherin or ARP2/3 antibody, respectively (top panel). Bottom panels show the positivity [total number of positive pixels/total number of pixels] that measured in RHGP (*n* = 5) and DHGP (*n* = 5) specimens using an optimized Aperio algorithm (mean + SD). C-RHGP Central tumor cells in RHGP lesions, P-RHGP Peripheral tumor cells in RHGP lesions, C-DHGP=Central tumor cells in DHGP, P-DHGP Peripheral tumor cells in DHGP lesions. Data are presented as the mean ± SD.

To address the role of RUNX1 in CRCLM vessel co-option, we examined the protein levels of RUNX1 in various CRCLM samples. It is worth mentioning that most of the CRCLM tumors are heterogeneous with a mixture of desmoplastic, replacement, and pushing^14,39^. Thus, the ratio of desmoplastic or replacement HGPs was quantified in each specimen by a histopathologist following the published consensus guidelines for scoring the HGPs^22^. Firstly, we evaluated the expression levels of RUNX1 in frozen sections of desmoplastic HGP and replacement HGP chemonaïve CRCLM lesions comparing to distal normal liver tissue. As shown in Fig. 1a, RUNX1 expression was increased in replacement lesions comparing to desmoplastic lesions. Interestingly, we noticed that the lesion with a higher percentage of replacement HGP expressed higher levels of RUNX1. Next, we used immunohistochemical (IHC) staining to further validate our results, which again demonstrated significant overexpression of RUNX1 in cancer cells of replacement lesions compared to desmoplastic (Fig. 1b). Positive RUNX1 staining was quantified using Aperio software^11,12,40^. Similar results were demonstrated in CRCLM specimens from patients treated with combined chemotherapy and bevacizumab (chemo + bev) as shown in Supplementary Fig. 1a. Interestingly, RUNX1 expression is uniformly higher in the cancer cells adjacent to liver tissue. To gain insight into RUNX1 biology, we investigated the expression of various genes, that have been reported to be transcriptionally regulated by RUNX1, in CRCLM lesions using our RNA-seq data that was recently published (GSE151165)^40^, as shown in Fig. 1c. We noticed that the majority of RUNX1 target genes were upregulated in replacement type CRCLM sections. The genes linked to EMT (e.g., *CDH2, CDH16, SNAI1, SNAI2*, *and VIM*)^41–43^ and cell motility (*ARPC1b, ARPC2*, *and ARPC3*)^44^ are among RUNX1 target genes that were upregulated in replacement lesions. However, the upregulation of some of these genes was not statistically significant. Thus, to validate some of these genes we stained CRCLM sections for E-Cadherin or ARP2/3. In-vessel co-opting lesions, we observed low expression of E-Cadherin in the cancer cells adjacent to liver tissue in chemonaïve and chemo+bev samples (Fig. 1d and Supplementary Fig. 1b). Conversely, the expression levels of ARP2/3 were almost two-fold higher in the cancer cells at the periphery of vessel co-opting lesions comparing to their counterparts in angiogenic lesions (Fig. 1e). Contrary to RUNX1 expression, we did not demonstrate any major difference in the expression of ARP2/3 between central and peripheral tumors in co-opted lesions. These observations suggest that although RUNX1 is a transcriptional factor of ARP2/3^31–33^, there are other possible proteins that regulate ARP2/3 expression that need to be explored in the future. Of note, previous investigations reported that low expression of E-cadherin accompanied by high expression of ARP2/3 contribute to EMT^45^ and motility^46^ in the cancer cells. Altogether, our data suggested a possible positive association of vessel co-option with RUNX1 and its target genes.

### TGFβ1 signaling through TGFβRII is required for RUNX1 expression

**Fig. 2:**
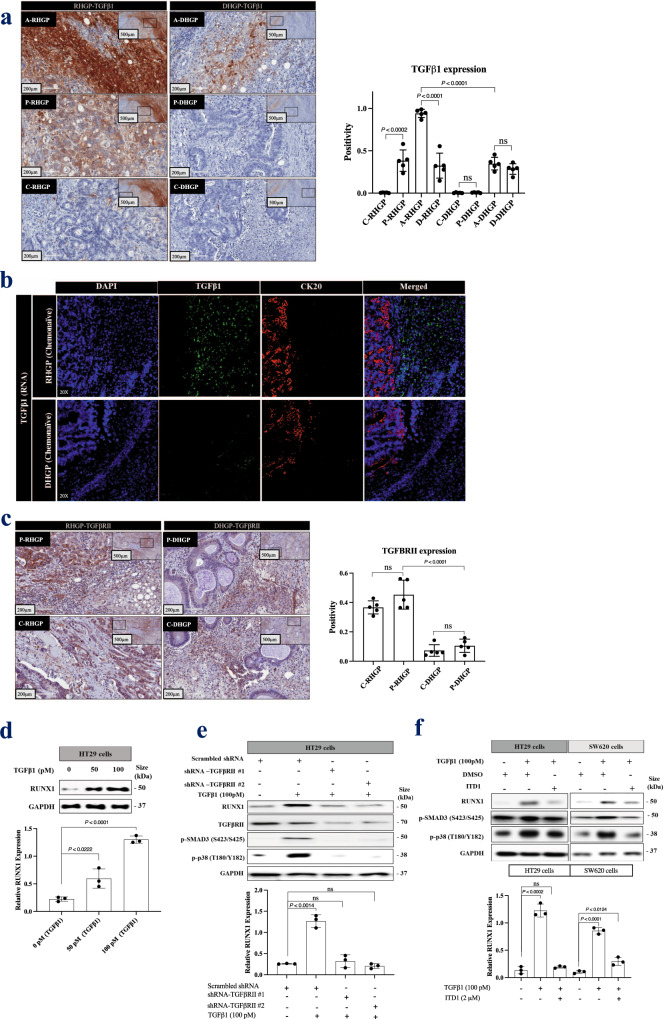
TGFβ1 regulates RUNX1 expression in cancer cells through TGFβRII. **a** Left panel represents immunohistochemical staining of RHGP (*n* = 5) and DHGP (*n* = 5) chemonaïve CRCLM specimens using TGFβ1 antibody. The right panel shows the positivity [total number of positive pixels/total number of pixels] was measured using an optimized Aperio algorithm (mean + SD). **b**. Fluorescence in situ hybridization (FISH) for TGFβ1 mRNA (green) expression in chemonaïve CRCLM lesions overlapped with CK20 (cytokeratin 20, red) antibody. **c** The left panel shows immunohistochemistry staining of chemonaïve CRCLM lesions with TGFβRII antibody. The right panel shows the positivity [total number of positive pixels/total number of pixels] was measured using an optimized Aperio algorithm (mean + SD). **d** Western blot of RUNX1 in HT29 cancer cells upon treatment with recombinant TGFβ1 for 24 h (top panel). **e** Western blot of RUNX1 and TGFβRII in HT29 cancer cells expressing either scrambles shRNA or shRNA against TGFβRII in the presence or absence of recombinant TGFβ1 for 24 h (top panel). **f** Western blot of RUNX1 in HT29 and SW620 cancer cells in the presence or absence of recombinant TGFβ1 individually or TGFβ1 with 2 μM of TGFβRII inhibitor (ITD1) for 24 h (top panel). The bottom panels represent the intensity of the bands (*n* = 3). C-RHGP Central tumor cells in RHGP lesions, P-RHGP Peripheral tumor cells in RHGP lesions, A-RHGP Adjacent hepatocytes to tumor lesion in RHGP, D-RHGP Distal hepatocytes to tumor lesion in RHGP, C-DHGP Central tumor cells in DHGP, P-DHGP Peripheral tumor cells in DHGP lesions, A-DHGP Adjacent hepatocytes to tumor lesion in DHGP, D-DHGP Distal hepatocytes to tumor lesion in DHGP. Data are presented as the mean ± SD.

Various studies reported RUNX1 as a mediator of Transforming Growth Factor Beta (TGFβ) family function^47–49^. Therefore, we hypothesized that TGFβ1 may regulate RUNX1 expression in CRCLM. To investigate the role of TGFβ1, we analyzed the expression of TGFβ1 in CRCLM sections. Our immunohistochemical staining results from both chemonaïve patients and patients that were treated with chemo + bev showed significant elevation in TGFβ1 expression in vessel co-opting type CRCLM lesions relative to desmoplastic (Fig. 2a and Supplementary Fig. 2a). Importantly, TGFβ1 staining was significantly higher (*p* < 0.0001) in hepatocytes at the tumor-liver interface where the hepatocytes of the normal adjacent liver and cancer cells in the replacement HGP are in very close proximity. To identify unequivocally in which tissue compartment the expression of TGFβ1 is upregulated, we performed fluorescence in situ hybridization (FISH) assay for TGFβ1 combined with cancer cell-specific (anti-Cytokeratin 20) staining. The results demonstrated that TGFβ1 is mainly expressed in the adjacent normal liver parenchyma (most likely hepatocytes) in CRCLM lesions and not in the cancer cells (Fig. 2b). We further confirmed these results using the FISH assay for TGFβ1 combined with Hepatocyte Specific Antigen (HSA) staining (Supplementary Fig. 2b). Accordingly, any positive staining of adjacent cancer cells for TGFβ1 is most likely due to their uptake of the secreted TGFβ1 from the liver parenchyma (hepatocytes) rather than an upregulated expression of TGFβ1 in cancer cells.

We also demonstrated upregulation of the TGFβ1 receptor II (TGFβRII) in chemonaïve replacement HGP tumors (Fig. 2c). SMAD2 and p38 represent canonical and non-canonical TGFβ1 signaling pathways, respectively^50^. We observed phosphorylated SMAD2 (S465/467) and phosphorylated p38 (T180/Y182) at the tumor-liver interface of RHGP lesions, but not at the tumor-liver interface of DHGP lesions (Supplementary Fig. 3). Remarkably, the adjacent hepatocytes of co-opted lesions also expressed high levels of phosphorylated SMAD2 (S465/467) and phosphorylated p38 (T180/Y182), indicating that TGFβ1 in the adjacent hepatocytes of co-opted lesions may participate in furnishing a favorable environment for cancer cells to establish vessel co-option via multiple mechanisms. These mechanisms might include inducing hepatocyte displacement by cancer cells^14,51^ or overexpression of Angiopoietin-1 (Ang1) in adjacent hepatocytes^12^. Our lab is currently examining these hypotheses.

Next, we asked whether RUNX1 expression in cancer cells was affected by the presence of TGFβ1 or TGFβRII in vitro. Firstly, we treated CRC (HT29) cells with recombinant TGFβ1. RUNX1 was strongly expressed upon exposure to TGFβ1 (Fig. 2d). This effect of TGFβ1 in RUNX1 expression was inhibited in the cancer cells expressing shRNA against TGFβRII (Fig. 2e). Similar results were reproduced using TGFβRII inhibitor (ITD1)^52^ in both HT29 and SW620 colorectal cancer cells (Fig. 2f). Taken together, these experiments indicate that TGFβ1 signaling can induce RUNX1 expression in cancer cells through TGFβRII.

### RUNX1 modulates TGFβ1 expression in hepatocytes through TSP1

**Fig. 3:**
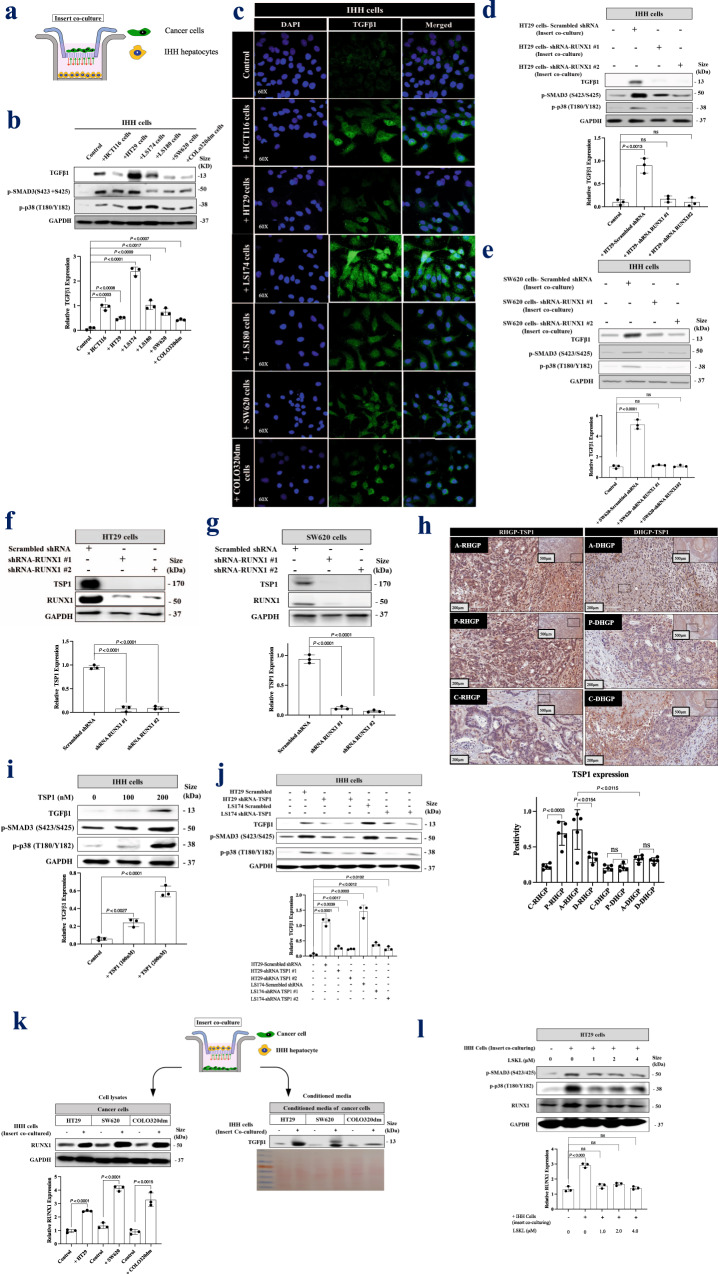
RUNX1 forms a positive feedback loop with TGFβ1 through TSP1. **a** Schematic of experimental design. **b** Western blot of TGFβ1 phosphorylated SMAD3 (S423/S425) and phosphorylated p38 (T180/Y182) in co-cultured IHH hepatocytes with different colorectal cancer cell lines. The bottom panel represents the intensity of TGFβ1 bands (*n* = 3). **c** TGFβ1 immunofluorescence staining of IHH hepatocytes co-cultured with various cancer cell lines. **d**, **e** Immunoblotting represents TGFβ1 expression in co-cultured IHH hepatocytes with HT29 or SW620 cancer cells expressing either shRNA-Scrambled or shRNA-RUNX1 (top panel). The bottom panel represents the intensity of TGFβ1 bands (*n* = 3). **f**, **g** Immunoblotting showing TSP1 expression in HT29 and SW620 cancer cells, respectively in the presence or absence of shRNA-RUNX1 (top panel). Bottom panels represent the intensity of TSP1 bands (*n* = 3). **h** The top panel shows immunohistochemical staining of chemonaïve CRCLM lesions with TSP1 antibody. The bottom panel represents quantification of TSP1 positivity [total number of positive pixels/total number of pixels] that measured in RHGP (*n* = 5) and DHGP (*n* = 5) lesions using an optimized Aperio algorithm (mean + SD). A-RHGP=Adjacent hepatocytes to tumor lesion in RHGP, C-RHGP=Central tumor cells in RHGP, P-RHGP=Peripheral tumor cells in RHGP, A-DHGP=Adjacent hepatocytes to tumor lesion in DHGP, C-DHGP=Central tumor cells in DHGP, P-DHGP Peripheral tumor cells in DHGP. **i** Immunoblotting shows TGFβ1 expression in the IHH hepatocyte cell line upon TSP1 treatment (top panel). The bottom panel represents the intensity of TGFβ1 bands (*n* = 3). **j** Immunoblotting shows TGFβ1 expression in co-cultured IHH hepatocytes with colorectal cancer (HT29 and LS174) cells expressing either shRNA-Scrambled or shRNA-TSP1 (top panel). The intensity of the bands was quantified using ImageJ and represented as a fold change (bottom panels), *n* = 3 independent experiments. **k** Schematic of experimental strategy (top panel). The bottom left panel shows the Western blot of RUNX1 in colorectal cancer (HT29, SW620, and COLO320dm) cells cultured either individually or co-cultured with hepatocyte (IHH) cell line. The intensity of the bands of RUX1 was quantified using ImageJ and represented as a fold change, *n* = 3 independent experiments. The bottom right panel is representative of a Western blot showing the abundance of TGFβ1 in the condition media of colorectal cancer (HT29, SW620, and COLO320dm) cells cultured either individually or co-cultured with IHH hepatocytes. Ponceau staining was used as a loading control. **l** Western blot of RUNX1, SMAD3 (S423/S425), and phosphorylated p38 (T180/Y182) in HT29 cells co-cultured hepatocyte (IHH) cells in the presence or absence of LSKL treatment (left panel). The bottom panel shows the intensity of RUNX1 bands (*n* = 3). Data are presented as the mean ± SD.

To identify the role of cancer cells in TGFβ1 expression by hepatocytes, we examined the expression of TGFβ1 in IHH hepatocytes in the presence or absence of cancer cells (HCT116, HT29, LS174, LS180, SW620, and COLO320dm) using insert co-culturing approach (Fig. 3a). IHH cells are immortalized human hepatocytes that retained several differentiated features of normal hepatocytes^53–55^. As shown in Fig. 3b, co-culturing hepatocytes with various colorectal cancer cell lines enhanced TGFβ1 expression in the hepatocytes. We further confirmed these results using immunofluorescence staining (Fig. 3c).

We speculated that the expression of TGFβ1 in hepatocytes could be induced by secreted factors from the cancer cells, which are under the transcription control of RUNX1. To test this hypothesis, we knocked down RUNX1 in HT29 and SW620 cancer cells followed by co-culturing with hepatocytes (IHH cells). As shown in Figs. 3d and 3e, the absence of RUNX1 in the co-cultured cancer cells resulted in lower expression of TGFβ1 in hepatocytes. These results suggest that RUNX1 plays an essential role in the crosstalk between cancer cells and hepatocytes through its target genes.

Thrombospondin-1 (TSP1) is a secreted protein, encoded by *THBS1*, which is a target gene of RUNX1^56,57^. Soto-Pantoja et al. have reported TSP1 as a regulator of TGFβ1 expression in TSP1-null mice that failed to express TGFβ1^58^. Other investigations have also identified TSP1 as an activator of TGFβ1^59^. Our results suggested a positive correlation between TSP1 and RUNX1 expression in HT29 and SW620 colorectal cancer cells (Figs. 3f and 3g). We then turned our attention to those RUNX1 target genes that are upregulated in RNA-seq data that are shown in Fig. 1c. We noticed that the expression of TSP1 (*THBS1*) is higher in replacement CRCLM tumors in comparison to their desmoplastic counterparts. In both chemonaïve (Fig. 3h) and chemo + bev CRCLM specimens, our IHC data suggested a significant increase of TSP1 on the protein levels in replacement co-opted lesions (Supplementary Fig. 4a).

CD36 is among TSP1 receptors^60–62^ that mediate TSP1-TGFβ1 interaction^63–65^. We confirmed that CD36 acts as a receptor of TSP1 in hepatocytes in vitro using co-immunoprecipitation assay (Supplementary Fig. 4b). Intriguingly, immunohistochemical results showed overexpression of CD36 in hepatocytes adjacent to the cancer cells of replacement lesions in chemonaïve CRCLM sections (Supplementary Fig. 4c). Therefore, we hypothesized that the crosstalk between RUNX1 and TGFβ1 may be mediated by TSP1. To address this, we first treated IHH hepatocytes with different concentrations of recombinant TSP1. Exposing IHH cells to recombinant TSP1 resulted in higher expression of TGFβ1 (Fig. 3i). The effect of TSP1 on TGFβ1 expression by hepatocytes was further ascertained by generating TSP1-silenced HT29 and LS174 colorectal cancer cells (Supplementary Fig. 4d) and co-culturing them with IHH cells. The cancer cells with silenced-TSP1 were failed in stimulating TGFβ1 expression in hepatocytes (Fig. 3j).

To further examine the molecular mechanisms explaining the signaling crosstalk between hepatocytes and cancer cells; we conducted a co-culturing experiment between hepatocytes and cancer cells followed by Western blotting to evaluate RUNX1 expression in the cancer cells. Co-culturing hepatocytes with colorectal cancer (HT29, SW620, and COLO320dm) cells resulted in a dramatic increase of RUNX1 protein levels (Fig. 3k, left panel). This might be due to the presence of TGFβ1 in the conditioned media of co-cultured cells (Fig. 3k, right panel). We repeated a similar experiment by co-culturing HT29 cancer cells with hepatocytes in the presence of LSKL (Leucine-Serine-Lysine-Leucine), an inhibitor of TSP1-mediated TGFβ1 activation^66,67^. The expression of RUNX1 was reduced to normal levels upon LSKL1 treatment (Fig. 3l). In summary, these data suggest signaling crosstalk between hepatocytes and cancer cells that regulates the expression of both TGFβ1 and RUNX1, which is orchestrated through TSP1.

### RUNX1 inhibition suppresses TGFβ1-driven EMT and motility in colorectal cancer cells in vitro

TGFβ1 plays a crucial role in colorectal cancer cells EMT and cell invasion^68^. To find out if RUNX1 can modulate TGFβ1 function in these processes, we performed various in vitro experiments and treated cancer cells with either recombinant TGFβ1 or co-cultured with hepatocyte (IHH) cells, upon RUNX1 inhibition.

RUNX1 binding to Core-Binding Factor Subunit Beta (CBFβ) has been reported, which stabilizes the RUNX-DNA interaction allosterically^69^. Interestingly, immunohistochemical staining showed positive staining for CBFβ in CRCLM sections as well (Supplementary Fig. 5a). Ro5-3335 inhibits CBFβ binding with RUNX1 which then blocks RUNX1 transcriptional activity^35,70^. Therefore, we used Ro5-3335 as a RUNX1 inhibitor for our in vitro experiments.

**Fig. 4:**
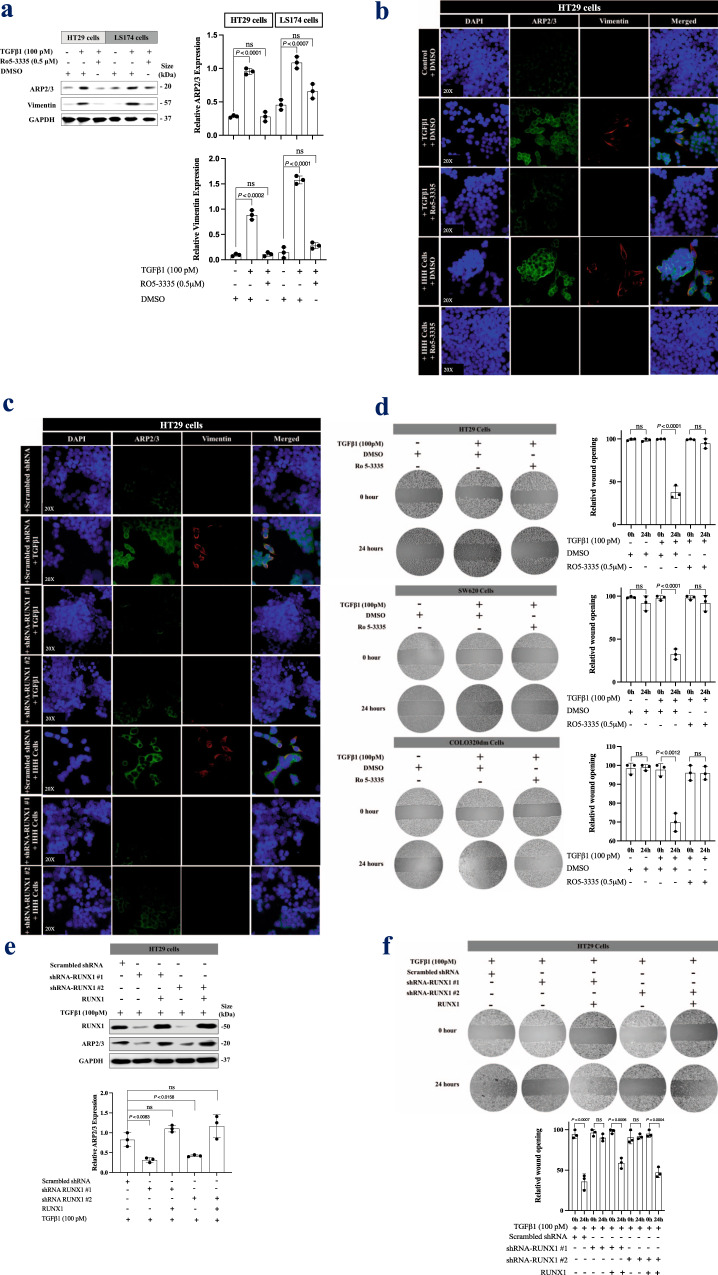
TGFβ1 promotes cancer cells motility through RUNX1. **a** Western blot of ARP2/3 and vimentin expression in HT29 or LS174 cells upon exposure to recombinant TGFβ1 (left panel). The right panel represents the intensity of ARP2/3 and vimentin bands (*n* = 3). **b** Immunofluorescence staining of colorectal HT29 cancer cells showing the effect of RUNX1 inhibitor (Ro5-3335, 0.5 μM) on the expression of ARP2/3 (green) and vimentin (red) in the presence of TGFβ1 (100pM) or co-cultured hepatocyte (IHH) cell line. **c** Immunofluorescence staining of ARP2/3 (green) and vimentin (red) in colorectal cancer (HT29) cells expressing either scrambled or RUNX1 shRNA. The cells were either treated with TGFβ1 (100pM) or co-cultured with a hepatocyte (IHH) cell line for 24 h. **d** Representative scratch assay in colorectal cancer (HT29, SW620, and COLO320dm) cells upon treatment with TGFβ1 (100pM) individually or combined TGFβ1 (100pM) plus RUNX1 inhibitor (Ro5-3335, 0.5 μM). **e** Immunoblotting showing ARP2/3 expression in RUNX1-silenced HT29 cancer cells expressing ectopic RUNX1 (top panel). The intensity of the bands was quantified using ImageJ and represented as a fold change. *n* = 3 independent experiments (bottom panel). **f** Scratch assay showing the rescue effects of RUNX1 in HT29 cancer cells invasion. Data are presented as the mean ± SD.

To identify the role of RUNX1 in TGFβ1-driven motility and EMT in colorectal cancer cells, we treated HT29 and LS174 colorectal cancer cells with TGFβ1 in the presence or absence of Ro5-3335 for 24 h followed by immunoblotting using anti-ARP2/3 and anti-vimentin antibodies representing cancer cell invasion^71^ and EMT^72^, respectively. As shown in Fig. 4a,

TGFβ1-dependent ARP2/3 and vimentin expression in cancer cells were attenuated in the presence of RUNX1 inhibitor (Ro5-3335). Similar results were obtained using immunofluorescence staining in HT29 (Fig. 4b) and COLO320dm cancer cells (Supplementary Fig. 5b). These data were further validated when RUNX1 function in HT29 cancer cells was suppressed using RUNX1 shRNA (Fig. 4c).

RUNX1 has been reported as a transcriptional factor for different genes including Ang1^73^ and IGFBP3^74^, encoding angiopoietin-1 and Insulin-like growth factor-binding protein-3 proteins, respectively. Both Ang1^75^ and IGFBP3^76^ are known as angiogenesis inhibitors. Therefore, we questioned whether RUNX1 and TGFβ1 are involved in the expression of these genes in CRC cells. Our immunofluorescence staining demonstrated upregulation of both proteins in CRC cells upon exposure to recombinant TGFβ1, while the effect of TGFβ1 was impaired in the presence of RUNX1 inhibitor (Ro5-3335) (Supplementary Fig. 6a and 6b). We recently published a paper^12^ demonstrating that knocking out of Ang1 in the host liver significantly induces the formation of desmoplastic CRCLM lesions. However, major gaps in our knowledge need fulfillment in future studies regarding the impact of Ang1 and/or IGFBP3 overexpression in cancer cells in the establishment of co-opted tumor lesions in the liver.

To evaluate the effect of RUNX1 in TGFβ1-mediated invasion in colorectal cancer cells, we conducted a wound-healing assay using three different colorectal cancer cell lines including HT29, SW620, or COLO320dm (Fig. 4d). We used recombinant TGFβ1 either individually or in combination with RUNX1 inhibitor (Ro5-3335) to treat the cancer cells for 24 h. The TGFβ1-treated cells showed higher levels of wound healing, while the presence of RUNX1 inhibitor suppressed this effect. Consistently, silencing RUNX1 in HT29 cells by shRNA attenuated the function of TGFβ1 in wound healing (Supplementary Fig. 6c). Importantly, the ectopic expression of RUNX1 restored both TGFβ1-dependent ARP2/3 expression (Fig. 4e) and wound healing (Fig. 4f) that were inhibited by RUNX1 depletion. Taken together, these results implied that proper RUNX1 function is necessary for TGFβ1 signaling in EMT and cell invasion in colorectal cancer cells.

### RUNX1 knockdown in cancer cells promotes the formation of angiogenic desmoplastic CRCLM lesions in vivo

**Fig. 5:**
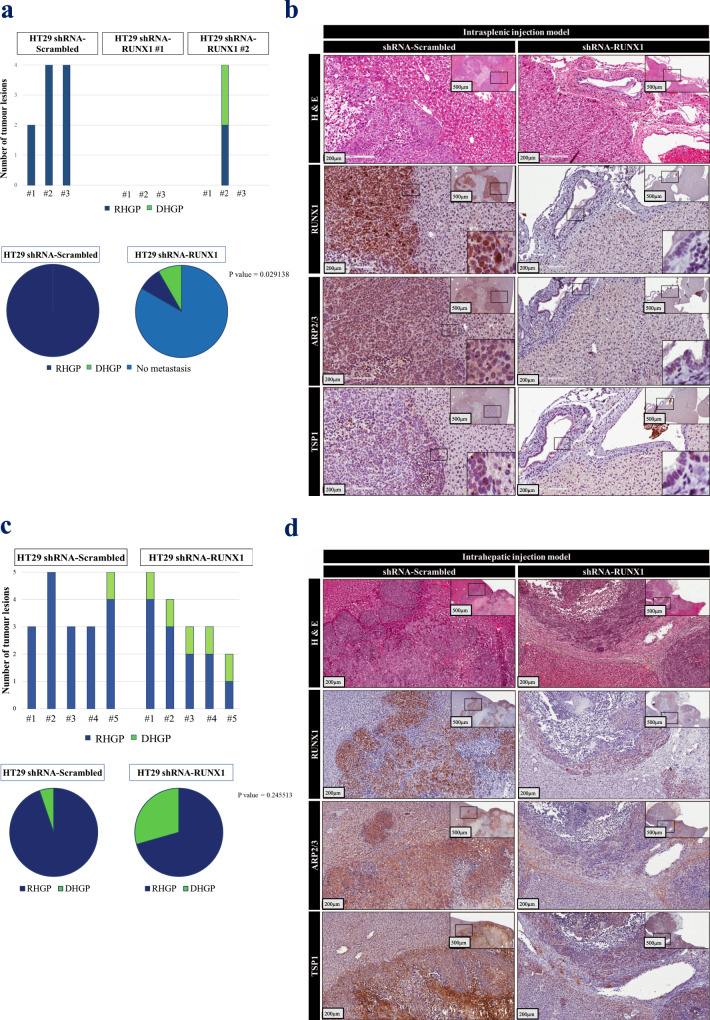
Silencing RUNX1 in CRC cells attenuated their capability for metastasis and development of co-opted lesions in vivo. **a** Represents number (top panel) and ratio (bottom panel) of hepatic tumor lesions that developed from intrasplenically injected mice with control or RUNX1-depleted HT29 cancer cells. *P* values were calculated using the Chi-square test. **b** Represents H&E and immunohistochemical staining of metastatic tumor sections. **c** The number (top panel) and ratio (bottom panel) of developed hepatic tumor lesions from intrahepatically injected mice by both control and RUNX1-depleted HT29 cells are shown. *P* values were calculated using the Chi-square test. **d** Represents H&E and immunohistochemical staining of tumor sections.

To gain a better understanding of the effects of RUNX1 in vessel co-option and identify its importance in developing or maintaining vessel co-opted CRCLM tumors in vivo, we used both intrasplenic and intrahepatic xenograft mouse models. Firstly, we injected SCID Beige mice intrasplenically with HT29 cancer cells expressing scrambled or RUNX1 shRNA. The RUNX1-deficient HT29 cancer cells showed lower capability for liver metastasis and formation of replacement vessel co-option lesions comparing to control (Figs. 5a and 5b). Accordingly, all the mice (3/3) that injected with control HT29 cells were developed liver metastasis, while 83% (5/6) of the mice that injected RUNX1-depleted HT29 cells lacked liver metastasis. Significantly, all developed metastatic lesions in the control group had replacement histological growth patterns, while 50% of the lesions in the one mouse that developed liver metastases in the shRNA-RUNX1 group were desmoplastic type. Immunohistochemical staining was used to determine the expression levels of RUNX1, ARP2/3, TSP1 (Fig. 5b, d), and TGFβ1 (Supplementary Fig. 7) in tumor sections, which we found lower levels of their expression in RUNX1-knockdown specimens than their control counterparts.

To further elucidate the role of RUNX1 in the development of vessel co-option in vivo, HT29 cancer cells expressing scrambled or RUNX1 shRNA were injected into SCID Beige mice intrahepatically. After 6 weeks, the tumors were analyzed for histopathological growth patterns. A dramatic increase in the ratio of desmoplastic lesions was detected in mice injected with RUNX1-depleted cancer cells comparing to controls (Figs. 5c and 5d). We also noticed a reduction in the size and number of lesions in the absence of RUNX1 in injected cancer cells. Since RUNX1 knockdown reduces cell proliferation in HT29 colorectal cancer cells (Supplementary Fig. 8), the size reduction in the RUNX1-depleted tumors may be caused by lower cell proliferation of the cancer cells. Overall, these results confirm the governing role of RUNX1 in the development or maintenance of the vessel co-opting replacement CRCLM lesions.

## Discussion

Colorectal cancer liver metastasis represents one of the most challenging tumors to treat, and these tumors are often resistant to anti-angiogenic therapy^10^. Different angiogenic and non-angiogenic compensatory pathways contribute to the adaptation of tumors to anti-angiogenic drugs. Vessel co-option^9^, increased pericyte coverage^77^, vasculogenic mimicry^78^, autophagy^79^, lysosomal sequestration^80^, and glycosylation-dependent^81^ resistance are among the mechanisms that may contribute to anti-angiogenic therapy resistance^10,82,83^. In CRCLM, we believe that vessel co-option is the main alternative vascularization pathway that could drive anti-angiogenic therapy resistance^11,12,14^. Of note, vessel co-option vascularization is primarily linked to replacement subtype CRCLM tumors^11,14^. The mechanistic pathways by which vessel co-option occurs in CRCLMs are poorly understood. Various studies reported that cancer cells in co-opted tumors are characterized by higher levels of motility^9,11,12,14,20,29^. In this context, a recent study from our lab demonstrated that knockdown of the ARP2/3 subunit ARPC3 attenuates vessel co-option in CRCLMs^14^. However, further investigation is required to identify the molecular pathways that regulate ARP2/3 expression, as well as the role of crosstalk between the normal liver and cancer cells in the replacement HGP.

The role of RUNX1 in angiogenesis is controversial to some extent. In this context, RUNX1 has been reported as a pro-angiogenic protein^84–87^. Mice deficient in RUNX1 die as embryos or soon after birth with a defect in definitive hematopoiesis^88^. On the other hand, various studies demonstrated RUNX1 blocks angiogenesis through repression of VEGF expression^89–91^. Moreover, Lirdprapamongkol et al.^92^ have reported RUNX1 as one of the molecules that upregulated in the tumor cells in vasculogenic mimicry lesions in hepatocellular carcinoma, while it is not clear how RUNX1 orchestrates the vasculogenic mimicry. Vasculogenic mimicry is a non-angiogenic phenotype where cancer cells mimic endothelial cells by forming blood vessel-like structures that are perfused via connection to the host vasculature^10^. In this study, we found the role of RUNX1 in vessel co-option. Our data suggested RUNX1 as an upstream transcriptional regulator of ARP2/3 in metastatic colorectal cancer cells in liver metastases. In agreement with our results, other studies suggested RUNX1 as a positive regulator of ARP2/3^31–33^, which controls the expression of various subunits including ARPC1, ARPC2, and ARPC3^34^. It is noteworthy that TGFβ family has been reported as a regulator of RUNX1^48,93–95^. TGFβ1 is among TGFβ members that modulate RUNX1 expression in cancer cells^96^. In agreement with these findings, we demonstrated an increase of RUNX1 expression upon TGFβ1 treatment.

TGFβRII is a well-established receptor for TGFβ1 that is associated with poor prognosis in various cancers, such as metastatic breast cancer^97^, colorectal cancer^98^, and prostate cancer^99^. Importantly, TGFβRII has been identified as an essential mediator of TGFβ1-dependent RUNX1 expression^100^. Similarly, we observed that the knockdown of TGFβRII diminishes the function of TGFβ1 towards RUNX1 expression in colorectal cancer cells.

**Fig. 6:**
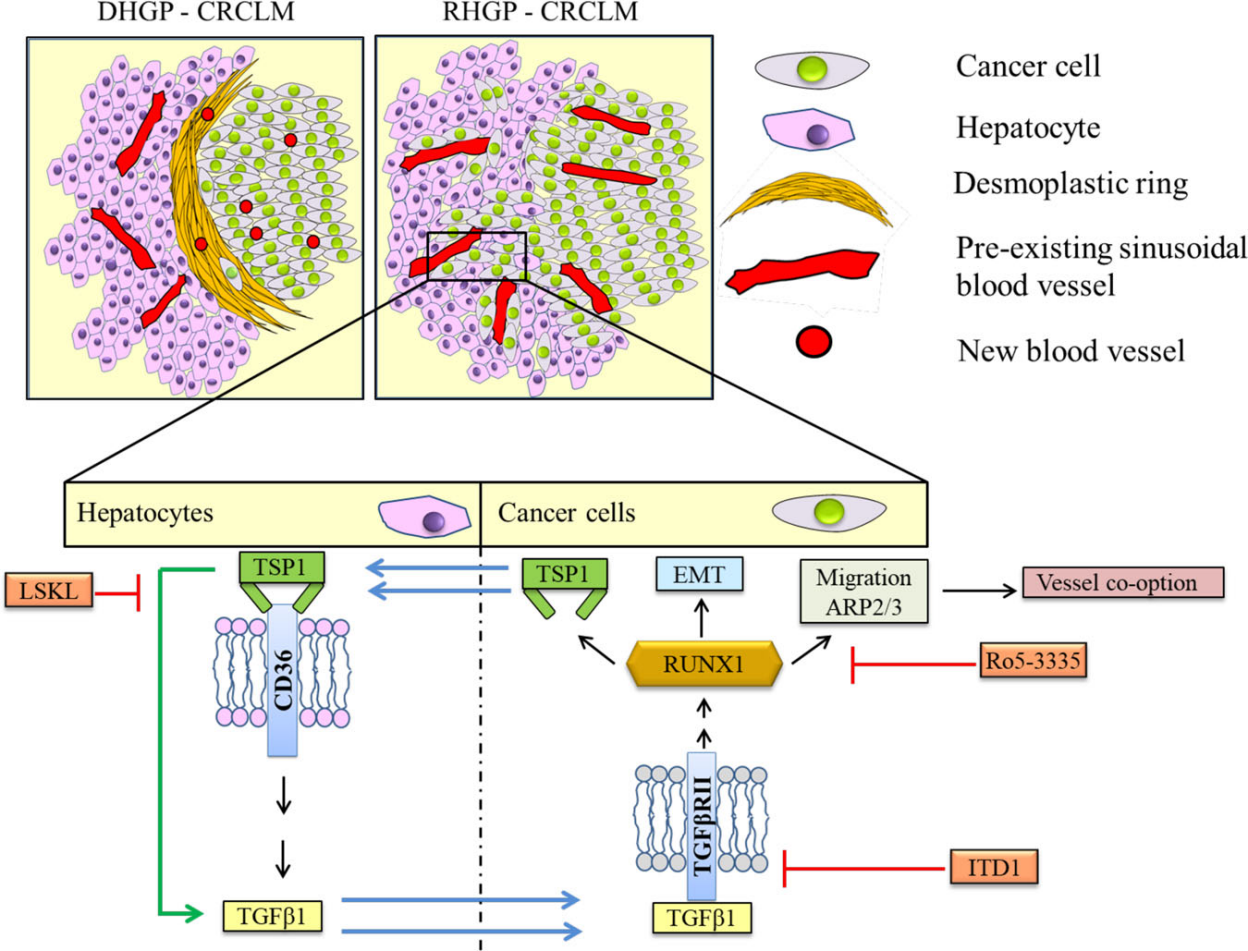
Proposed model of RUNX1 signaling in co-opted CRCLM lesions. Schematic representation of key findings in our study. RUNX1 plays a central role in the development of vessel co-option in CRCLM. RUNX1 overexpression results in the expression of its target genes that contribute to cancer cells motility and EMT. TSP1 is one of the RUNX1 target genes that are expressed and secreted by cancer cells, which modulates the expression and activity of TGFβ1 in the hepatocytes in the normal adjacent liver. The secreted TGFβ1 by adjacent hepatocytes contributes to RUNX1 overexpression in the peripheral cancer cells through TGFβRII. Consequently, RUNX1 forms a positive feedback loop with TGFβ1 through TSP1.

RUNX1 is known as a transcriptional regulator of *THBS1* that encodes the TSP1 protein^56,101^, which is highly expressed in replacement type CRCLM tumors (GSE151165)^40^. TSP1 is an anti-angiogenic protein^102,103^ that fulfills a plethora of biological functions and its overexpression is associated with invasive and metastatic phenotypes in various cancers, for instance, glioblastoma^29^, prostate cancer^104^, and medulloblastoma^105^. Pleiotropic effects of TSP1 are exerted by its binding to diverse receptors including CD47, CD36, LRP1, and integrin α3β1^106^. CD36 is among the TSP1 receptors involved in TSP1-dependent TGFβ1 expression and activation^63–65^. Our results showed overexpression of CD36 in the hepatocytes of replacement CRCLM lesions; specifically, those bordering the cancer cells. However, future experiments will be needed to gain information on other TSP1 receptors (e.g., CD47, LRP1, and integrin α3β1) and identify their role in CRCLM vessel co-option. Similar to CD36, high expression of TGFβ1 was found in the hepatocytes adjacent to cancer cells compared to distal hepatocytes. This phenomenon supports the possibility that TSP1 may be responsible for TGFβ1 upregulation in the hepatocytes of the co-opted CRCLM sections, and CD36 seems to play a key role in this process. Indeed, our in vitro data also confirmed the importance of TSP1 for TGFβ1 expression in hepatocytes. The outcome of this study suggests a positive feedback loop between TGFβ1 and RUNX1, mediated via TSP1 (Fig. 6).

In this study, we mainly focused on the role of RUNX1 in chemonaïve CRCLM patients. However, we also observed significant upregulation of RUNX1 in the cancer cells of replacement HGP CRCLM lesions that are typically resistant to the combination of chemotherapy with bevacizumab (Chemo + bev) (Supplementary Fig. 1a). These data indicate that RUNX1 likely contributes to therapy resistance in CRCLM patients. RUNX1 has been associated with anti-cancer therapy resistance through PI3-kinase/Akt pathways in Acute megakaryoblastic leukemia (AMKL) individuals without Down syndrome (non-DS-AMKL)^107^. Likewise, the aberrant elevation of Runt-related transcription factor 2 (RUNX2) positively correlated with anti-cancer resistance in various cancers, such as osteosarcomas, breast cancer, and pancreatic cancer^108–110^.

In this study, we discovered the role of RUNX1 in vessel co-option. Although we presume that these results are mainly due to reduced ARP2/3-dependent cancer cell invasion^14^, decreasing the expression of anti-angiogenic genes that are regulated by RUNX1 (e.g., Ang1 and IGFBP3) may have played a role as well. Therefore, more studies are needed to explore the role of anti-angiogenic genes regulated by RUNX1 in the development of vessel co-opting CRCLM tumors.

In conclusion, this study revealed that RUNX1 plays an essential role in the development of replacement pattern vessel co-opting CRCLM lesions through regulation of its downstream molecules including ARP2/3, vimentin, and TSP1, which facilitate cancer cell motility. Also, we discovered a positive feedback loop between TGFβ1 and RUNX1, mediated by TSP1, which possibly has a potential implication for new strategies to overcome resistance to anti-angiogenic therapy.

## Methods

### Patient samples

The study was conducted in accordance with the guidelines approved by McGill University Health Centre Institutional Review Board (IRB). Informed consent was obtained from all patients through the McGill University Health Centre (MUHC) Liver Disease Biobank. Surgical specimens were procured and released to the Biobank immediately after the pathologist’s confirmation of carcinoma and surgical margins.

### Cell cultures

Human colorectal cancer (HT29, LS174, LS180, SW620, COLO320dm) cell lines were a gift kindly supplied by Dr. Alex Alex Gregorieff (Cancer Research Program, McGill University). HCT116 and HEK293T packaging cells were kindly provided by Dr. Daniela Quail and Dr. Peter Siegel, respectively (Rosalind and Morris Goodman Cancer Research Centre, McGill University). IHH cells were a generous gift from Dr. Nabil G. Seidah at Montreal Clinical Research Institute (IRCM). The cells were cultured in DMEM (Wisent Inc., #319-005-CL) supplemented with 10% FBS (Wisent Inc., #085-150) and 1× penicillin/streptomycin (Wisent Inc., 450-201-EL). All cells were cultured at 37 °C with 5% CO_2_.

Cells were treated with various inhibitors including ITD1 (Tocris, #5068), LSKL1 (AnaSpec, #AS-60877), and Ro5-3335 (Milipore, #219506).

### Co-culture, treating cells with recombinant TGFβ1 or TSP1

Before experiments with recombinant TGFβ1 (Peprotech, # 100-21) or TSP1 (Sigma Aldrich, #ECM002-50UG), the cells were seeded in DMEM (Wisent Inc., #319-005-CL) supplemented with 10% FBS (Wisent Inc., #085-150) and 1× penicillin/streptomycin (Wisent Inc., 450-201-EL) overnight. The next day, the conditioned media was aspirated, and the cells were washed with PBS (Wisent Inc., #311-010-CL) twice. Then, serum-free DMEM supplemented with either TGFβ1 or TSP1 was added and the cells were incubated for 24 h at 37 °C. Co-culturing was conducted using 6-well inserts (Falcon, #353090) and companion plates (Falcon #353502). The cells were cultured with DMEM (Wisent Inc., #319-005-CL) supplemented with 10% FBS (Wisent Inc., #085-150) and 1× penicillin/streptomycin (Wisent Inc., 450-201-EL) overnight. The next day, the media was removed, and the cells were washed twice with PBS (Wisent Inc., #311-010-CL). New serum-free DMEM (Wisent Inc., #319-005-CL) was added and incubated at 37 °C for 24 h.

### Immunoblotting

Immunoblotting was performed following the previously published protocols^111–114^. Briefly, cells were washed once with 1× PBS, trypsinized, collected, and kept on ice. Cells were ruptured by passing through a syringe 10 times and centrifuged for 10 min at 5000 rpm. The supernatant was transferred into 1.5 mL microcentrifuge tubes, and protein concentrations were determined using BCA Protein Assay Kit (Thermo Scientific, #23225). 5–10 μg of total protein per sample were subjected to 10–12% SDS-PAGE and transferred to Immobilon-E membranes (Millipore, #IEVH85R). The blots were developed using Pierce ECL Western Blotting Substrate (Thermo Scientific, #32106) and imaged with ImageQuant LAS4000 (GE Healthcare BioScience).

Lysate from frozen CRCLM tumor lesions was prepared by chopped the tumor tissues into small pieces and transferred them to a tube containing lysis buffer (100 mM Tris-HCl pH 8.0, 150 mM NaCl, 1% Triton X-100, 2 mM EDTA, 1 mM PMSF, protease inhibitor cocktail and double-distilled H_2_O). The cells were homogenized on ice using a tissue grinder and incubated for 30 min on ice. The samples were spun down at 15000 × *g* for 30 min and the supernatant was transferred to a fresh tube and another centrifuge was performed at 15,000 × *g* for 30 min. The supernatant was transferred to a fresh tube and used for Western blotting following the protocol. The intensity of the bands was measured using ImageJ (NIH, Bethesda, MD) software^111,115^. The uncropped blot images are shown in Supplementary Fig. 9.

The following primary antibodies were used: GAPDH 1:2000 (Abcam, #ab9485), TGFβ1 1:200 (Santa Cruz, #sc-130348), Phosphorylated p38 (Thr180/Tyr182) 1:500 (Cell Signaling Technology, #4631), Phosphorylated SMAD3 (Ser423/425) 1:1000 (abcam, #ab52903), RUNX1 1:500 (LS Bio, #LS-C353932), ARP2/3 1:1000 (Millipore, #MABT95), Vimentin 1:1000 (abcam, ab16700), TGFBRII 1:1000 (Thermo Fisher, #PA5-35076), Ang1 1:1000 (abcam, #ab102015) and IGFBP3 1:1000 (Proteintech, #10189-2-AP).

### Immunohistochemical staining

Formalin-fixed paraffin-embedded (FFPE) CRCLM resected blocks were used for this study. Serial sections 4 mm thick were cut from each FFPE block, mounted on charged glass slides (Fisher Scientific, #12-550-15), and baked at 37 °C overnight. Prior to staining, the slides were baked at 60 °C for 1 h as well. Hematoxylin and eosin (H&E)-stained sections were prepared from all cases for an initial histopathological assessment. The sections were deparaffinized with xylene (Leica, #3803665) followed by hydration with graded concentrations of ethanol (Comalc, #P016EAAN) and then with distilled water. Samples were subjected to antigen retrieval followed by washing with PBS and incubation in hydrogen peroxide (Dako, #S2003) to inhibit endogenous peroxidase. The tissue sections were blocked with 1% goat serum and incubated with the indicated primary antibody in 1% goat serum overnight at 4 °C. After washing, the sections were incubated with secondary antibody (Dako, Anti-Mouse: #K4001; Anti-Rabbit: #K4003) for 1 h at room temperature and positive signals were visualized with the diaminobenzidine (DAB) substrate (Dako, #K3468). The following primary antibodies were used: TGFβ1 1:1500 (Abcam, #ab27969), TGFβ1 1:100 (Abcam, #ab215715), RUNX1 1:200 (LS Bio, #LS-C353932), E-Cadherin 1:200 (R&D systems, #MAB1838-100), TGFBRII 1:200 (Thermo Fisher, #PA5-35076), CD36 1:200 (abcam, #ab133625), TSP1 1:200 (abcam, #ab1823), CBFβ 1:100 (LSBio, #LS‑C342588), ARP2/3 1:300 (Millipore, #MABT95; Bioss, #bs-12524R) and Ang1 1:50 (Abcam, #ab215715).

All slides were scanned at ×20 magnification using the Aperio AT Turbo system. Images were viewed using the Aperio ImageScope ver.11.2.0.780 software program for scoring analysis and assessment of signals. The positivity [Total number of positive pixels divided by the total number of pixels: (NTotal – Nn)/(NTotal)] was assessed with an Aperio ScanScope (Aperio Technologies Inc., Vista, CA)^11,12^.

### Immunofluorescence staining

Formalin-fixed paraffin-embedded (FFPE) human CRCLM resected blocks were deparaffinized with xylene followed by hydration with graded concentrations of ethanol and then with distilled water. Samples were subjected to antigen retrieval followed by washing with PBS and incubation in hydrogen peroxide (Dako, #S2003) to inhibit endogenous peroxidase. The tissue sections were blocked with 1% goat serum and incubated with the indicated primary antibody in 1% goat serum overnight at 4 °C. After washing, the sections were incubated with secondary antibody 1:1000 (Alexa Flour 594 goat anti-rabbit IgG and Alexa Flour 488 goat anti-mouse IgG (Invitrogen #A11037 and #A10680, respectively)) for 1 h at room temperature followed by washing thrice. The sections were incubated with 4′,6-Diamidino-2-Phenylindole, Dihydrochloride DAPI 1:1000 (Thermo Fisher Scientific, D1306) in PBS for 10 min at room temperature. Prior to mounting undercover glasses, 1–2 drops of ProLong Gold Antifade Mountant (Thermo Fisher Scientific, P36934) were added to each section.

Immunofluorescence staining for cells was performed following the protocol^115–117^. Briefly, the cells were fixed with 4% paraformaldehyde (Biolegend, #420801), washed with PBS, and permeabilized with 0.1% Triton X-100 (Bio-Rad, #161-0407).

The cells were then washed with PBS and blocked with 1% BSA (GE Healthcare Life Science, #SH30574.02) followed by incubation with primary antibody at 4 °C overnight. The following day, cells were washed with PBS and incubated with 100 μl of 1:1000 secondary antibodies for 1 h in the dark. After incubation, coverslips were washed three times with PBS and stained with DAPI 1:1000 for 10 min. The coverslips were mounted on slides using ProLong Gold Antifade Mountant (Thermo Fisher Scientific, P36934). Slides were visualized using a Zeiss LSM780 confocal microscope system. The following primary antibodies were used: TGFβ1 1:1500 (Abcam, #ab27969), Phospho-p38 1:50 (Thr180/Tyr182) (Cell Signaling Technology, #4631), TSP1 1:200 (abcam, #ab1823), ARP2/3 1:300 (Millipore, #MABT95), Vimentin 1:200 (abcam, ab16700), Phospho-Smad2 1:200 (Ser465/467) (Cell Signaling Technology, #3101), Cytokeratin 20 1:100 (abcam, #ab76126), Ang1 1:1000 (abcam,#ab102015), IGFBP3 1:200 (Proteintech, #10189-2-AP) and HSA 1:300 (Santa Cruz, #SC5893).

### Fluorescence in situ hybridization (FISH)

To identify TGFβ1 expression in CRCLM lesions fluorescence in situ hybridization was performed according to the manufacturer’s recommendations using RNAscope Probe-Hs-TGFβ1, labeled with Alexa 488 nm fluorescent dye^12^. Briefly, formalin-fixed paraffin-embedded (FFPE) human CRCLM sections (4 µm) were baked for 1 h at 60 °C. The sections were deparaffinized through successive baths of xylene (100%), ethanol (95%), and then distilled water. After drying, the slides were incubated for 10 min with RNAscope Hydrogen Peroxide at room temperature followed by washing. Then, target retrieval was conducted by incubating the slides with RNAscope 1× Target Retrieval Reagents in a steamer for 20 min. The sections were incubated with ethanol for 3 min, dried, and incubated with RNAscope Protease Plus at Incubate at 40 °C for 30 min. The slides were washed, dried, and Hybridization was carried out with RNAscope Probe-Hs-TGFβ1-C2 (ACDBIO, #400881-C2) diluted in a Blank Probe–C1 (ACDBIO, #300041) and incubated in HybEZ™ Oven (ACDBIO, #321710) at 40 °C for 2 h. The slides were then incubated with SSC buffer (Sigma-Aldrich/MLS, #S6639-1L) overnight at room temperature. The next day, the slides were washed and incubated at 40 °C with RNAscope Multiplex FL V2 AMP-1 (ACDBIO, #323110) for 30 min, RNAscope Multiplex FL V2 AMP-2 for 30 min and RNAscope Multiplex FL V2 AMP-3 for 15 min. After washing, the sections were incubated with RNAscope Multiplex FL v2 HRP-C1 (ACDBIO, #323110) for 15 min at 40 °C. The dye was prepared by diluting Opal 520 Reagent in RNAscope Multiplex TSA Buffer 1:1500 (ACDBIO, #322809) and added to the sections for 30 min at 40 °C followed by incubation with RNAscope® Multiplex FL v2 HRP blocker for 15 min at 40 °C. Next, we incubate the slides in 1% BSA for 30 min at room temperature and staining was performed with Cytokeratin-20 1:100 (abcam, #ab76126) following the abovementioned Immunofluorescence staining protocol. The sections were mounted under coverslip using ProLong Gold Antifade Mountant (Thermo Fisher Scientific, P36934) and visualized with Zeiss LSM780 confocal microscope system.

### Lentiviral shRNA knockdown

RUNX1, TSP1 TGFβRII knockdown was achieved using lentiviral shRNA vectors from the Mission TRC genome-wide shRNA collections purchased from Sigma-Aldrich Corporation with the following catalog numbers; Scrambled shRNA#: SHC016, RUNX1#1: TRCN0000338428, RUNX1#2: TRCN0000338427, TSP1#1: TRCN0000226402, TSP1#2: TRCN0000219072, TGFβRII#1: TRCN0000000831 and TGFβRII#2: TRCN0000000834. Lentiviral supernatants were generated using the calcium phosphate method as described^118^. Cancer cells were incubated with lentivirus-containing media with polybrene (8 µg/ml) and incubated for 72 h at 37 °C with 5% CO_2_ followed by 1 µg/ml of Puromycin (Wisent Inc., 450-162-XL) selection for 15 days.

### Co-immunoprecipitation (Co-IP)

Co-immunoprecipitation (Co-IP) was conducted following the protocol^115,117^. Hepatocyte (IHH) cancer cells were cultured at 37 °C for 24 h in DMEM media supplemented with 10% FBS and 1% 1× penicillin/streptomycin. The next day, the media was replaced with new serum-free DMEM media supplemented with recombinant TSP1 (Sigma Aldrich, #ECM002-50UG) for 24 h. The treated cells were collected and lysed. The extract solution was divided into three parts as follows: 10% as input, 45% for immunoprecipitation with anti-IgG antibody (Santa Cruz, #sc-2025), and 45% for immunoprecipitation with anti-CD36 antibody (abcam, #ab133625). 1 µg of the desired antibody was added to the extract solution and incubated overnight at 4 °C in the rotator. Concurrently, the beads (Millipore, #16-157) were blocked by mixing with 5% BSA and incubating overnight at 4 °C with rotation. The next day, the blocked beads were incubated with the lysate-antibody mixture for 4 h at 4 °C with rotation. Bound proteins were analyzed by Western blotting.

### Scratch assay

Before the experiments, the plates were coated with poly-L-Lysine (Millipore, #A-005-CL) and incubated for 30 min at 37 °C, followed by aspiration and air-drying. The cancer cells were seeded overnight using DMEM (Wisent Inc., #319-005-CL) supplemented with 10% FBS (Wisent Inc., #085-150) and 1× penicillin/streptomycin (Wisent Inc., 450-201-EL). The media was aspirated, and a wound was introduced into the monolayer cells using a p200 pipette tip. After washing with PBS (Wisent Inc., #311-010-CL), the denuded areas were photographed (0 h). Cells were then cultured using serum-free media for 24 h at 37 °C. The cells were washed, and the scratched areas were photographed (24 h)^12^. The relative wound opening was assessed using ImageJ (NIH, Bethesda, MD) software^119,120^.

### Proliferation assay

We performed this assay to determine the effect of RUNX1 on proliferation rates in HT29. A similar number of HT29 cells expressing either shRNA-Scramled or shRNA-RUNX1 were cultured in DMEM (Wisent Inc., #319-005-CL) supplemented with 10% FBS (Wisent Inc., #085-150), 1× penicillin/streptomycin (Wisent Inc., 450-201-EL) and 1 µg/ml of Puromycin (Wisent Inc., 450-162-XL) at 37 °C. Every 12 h the cells were collected by trypsinization followed by counting using trypan blue (Bio-Rad, #1450021).

### Xenograft experiments

To identify the role of RUNX1 on the histological growth pattern of CRCLM, we performed both intrasplenic and intrahepatic mouse model experiments. The mice were randomly assigned to each group. Colorectal cancer liver metastases were generated in 4-week to 6-week old SCID Beige mice by intrasplenic injection of 50 µL of PBS (Wisent Inc., #311-010-CL) containing 1 × 10^6^ HT29 shRNA-Scrambled, HT29 shRNA-RUNX1 #1, or HT29 shRNA-RUNX1 #2 followed by splenectomy 1 min after injection^12^. Mice were euthanized 6 weeks later. We also conducted intrahepatic injection to further validate the role of RUNX1 in vivo. 1 × 10^6^ HT29 (Scrambled or shRNA-RUNX1) colorectal cancer cells were injected into the liver of 4-week to 6-week old SCID Beige mice. All animals were monitored daily for survival until the experimental endpoint. After 6 weeks, the mice were sacrificed.

Next, sections of the liver were collected and fixed in 10% buffered neutral formalin, and paraffin-embedded. Hematoxylin and eosin (H&E)-stained sections were prepared from all samples for an initial histopathological assessment. The mice were housed in facilities managed by the McGill University Animal Resources Centre. All animal experiments were conducted under a McGill University-approved Animal Use Protocol in accordance with guidelines established by the Canadian Council on Animal Care.

### Statistical reproducibility

Statistical analysis was performed with a two-tailed Student’s *t*-test using GraphPad Prism software version 7.0 (GraphPad Software, La Jolla, CA, USA) and Excel software. Data presented as mean ± standard deviation. Unpaired Student’s *t*-test was applied to compare the means of two groups. The association between the two categorical groups in xenograft experiments was assessed with the Chi-square test. *P*-values of <0.05 were considered to be significant.

### Reporting summary

Further information on research design is available in the Nature Research Reporting Summary linked to this article.

## Supplementary information

Supplementary Information

Description of Supplementary Files

Supplementary Data 1

Reporting Summary

## Data Availability

RNA-seq data are publicly available in GEO with the accession (GSE151165). The datasets generated and/or analyzed during this study can be found in Supplementary Data 1. All other relevant data are available from the corresponding author upon reasonable request.
