# Supplementary material for: Runt related transcription factor-1 plays a central role in vessel co-option of colorectal cancer liver metastases
Source: Commun Biol. 2021 Aug 10;4:950. doi: 10.1038/s42003-021-02481-8 (PMC8355374; doi:10.1038/s42003-021-02481-8)
Supplement: Supplementary file 1 — Supplementary Information [file 42003_2021_2481_MOESM1_ESM.pdf]

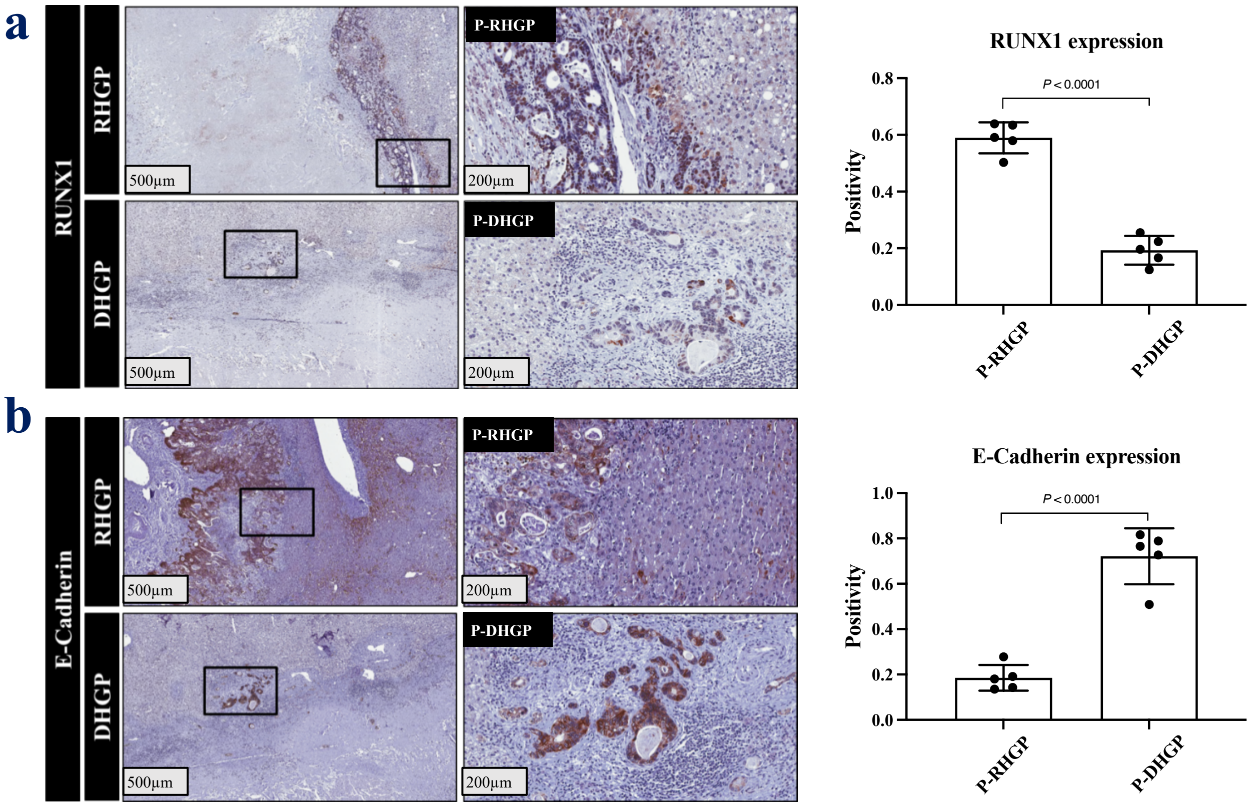

Rada *et al*., Supplementary Figure 1

Supplementary Figure 1. Expression of RUNX1 and E-Cadherin in CRCLM samples chemotherapy and bevacizumab (chemo+bev). a and b. Immunohistochemistry staining of CRCLM lesions with RUNX1 or E-cadherin antibody (left panel). Right panels represent the positivity [total number of positive pixels/total number of pixels] of RUNX1 or E-Cadherin staining in RHGP (n=5) and DHGP (n=5) that measured using an optimized Aperio algorithm (mean + SD). P-RHGP=Peripheral tumour cells in RHGP lesions, P-DHGP=Peripheral tumour cells in DHGP lesions.

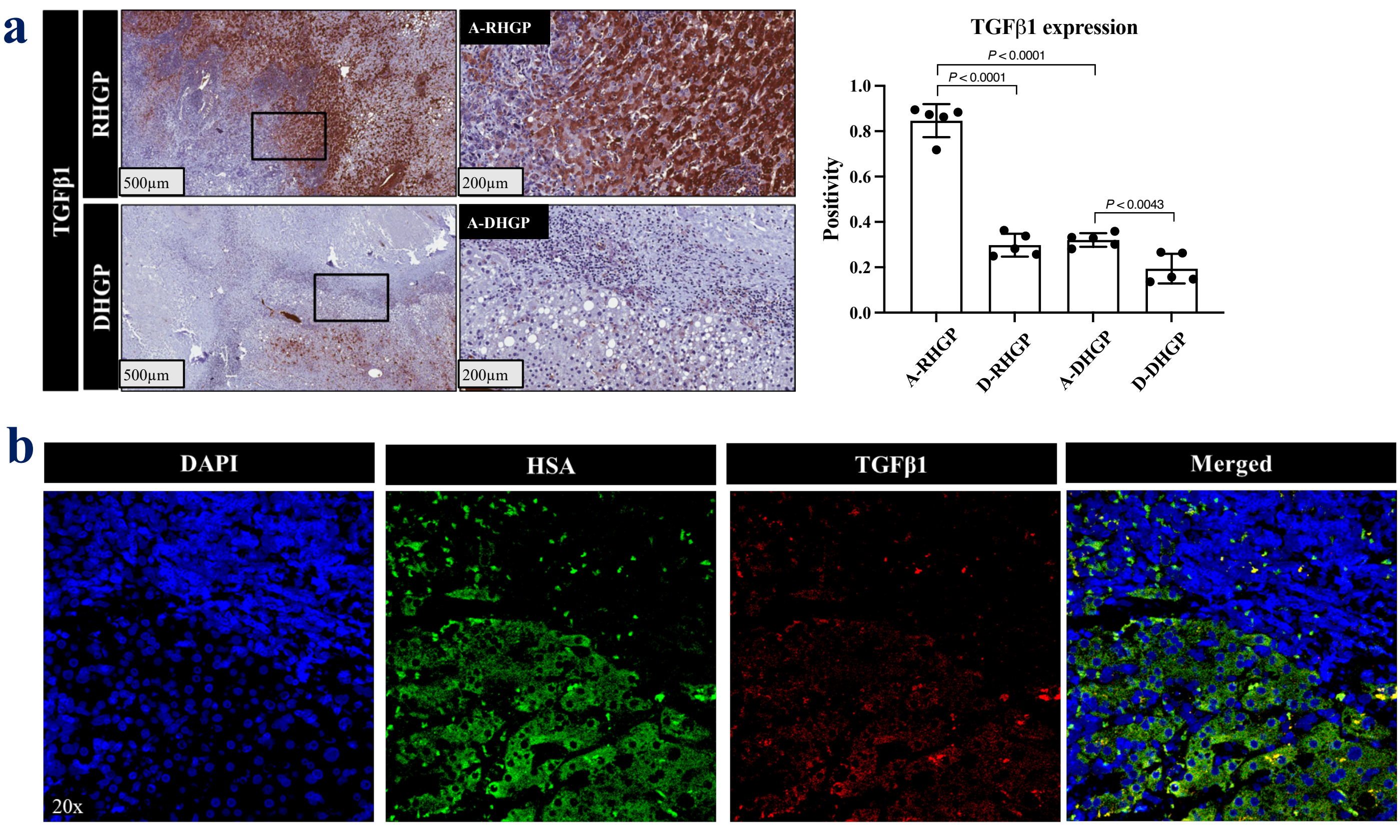

Rada *et al.*, Supplementary Figure 2

Supplementary Figure 2. The expression of TGFβ1 in CRCLM specimens mainly located in the liver parenchyma. a. Immunohistochemistry staining of CRCLM lesions (chemo+bev) with TGFβ1 antibody (left panel). Right panel shows the positivity [total number of positive pixels/total number of pixels] was measured using an optimized Aperio algorithm (mean + SD). A-RHGP=Adjacent hepatocytes to tumour lesion in RHGP, D-RHGP= Distal hepatocytes to tumour lesion in RHGP, A-DHGP=Adjacent hepatocytes to tumour lesion in DHGP, D-DHGP= Distal hepatocytes to tumour lesion in DHGP. **b.** Fluorescence in situ hybridization (FISH) for TGFβ1 mRNA (red) expression in chemonaïve CRCLM lesions overlapped with Hepatocyte Specific Antigen (HSA, green) antibody.

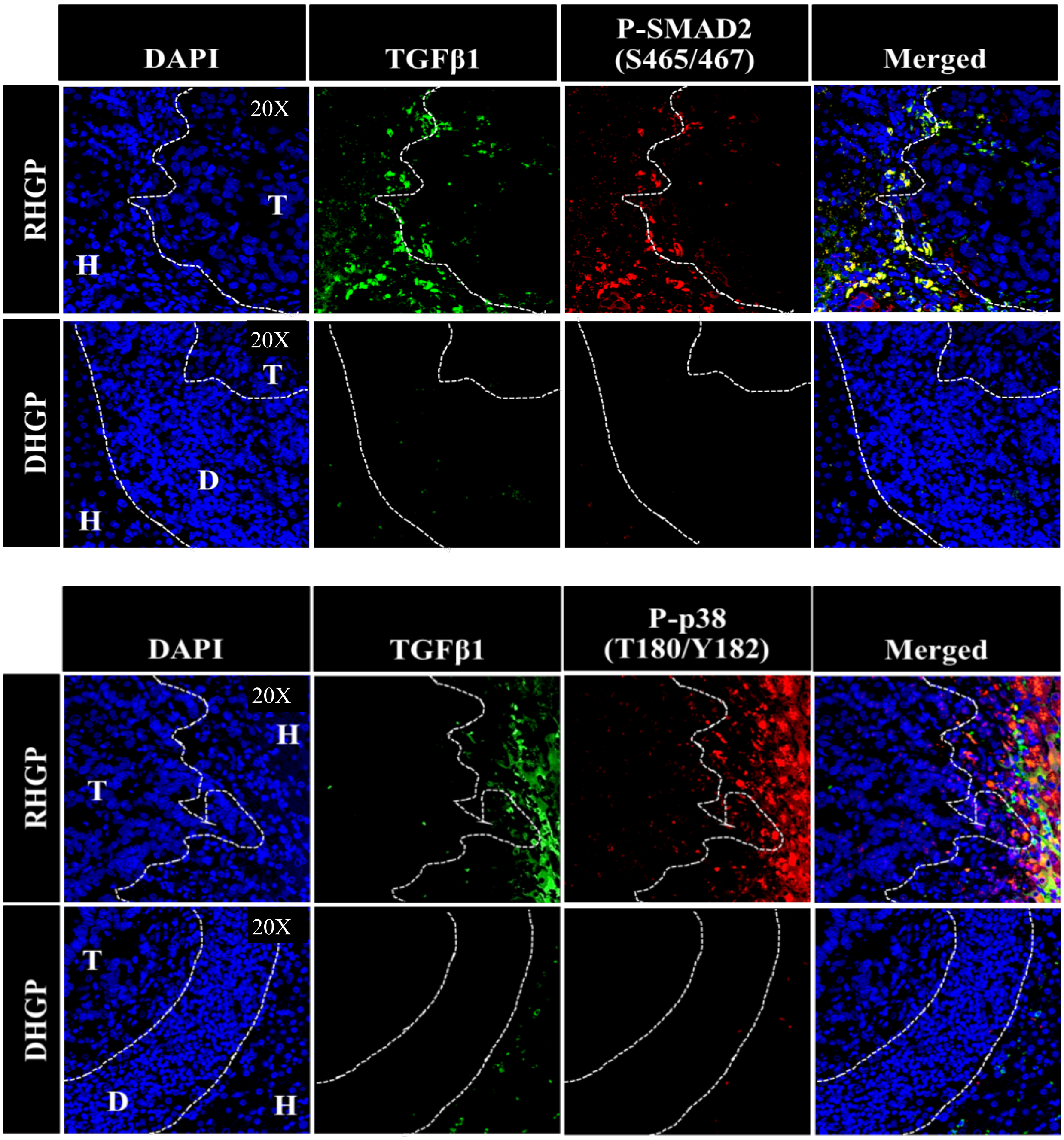

Rada *et al.*, Supplementary Figure 3

Supplementary Figure 3. The expression of canonical and non-canonical pathway markers in CRCLM lesions. Immunofluorescence staining of chemonaïve CRCLM lesions showing TGFβ1 (green) and phosphorylated SMAD2 (S465/S467) or phosphorylated p38 (T180/Y182) (red). T: Tumour; H: Hepatocytes; D: Desmoplastic ring.

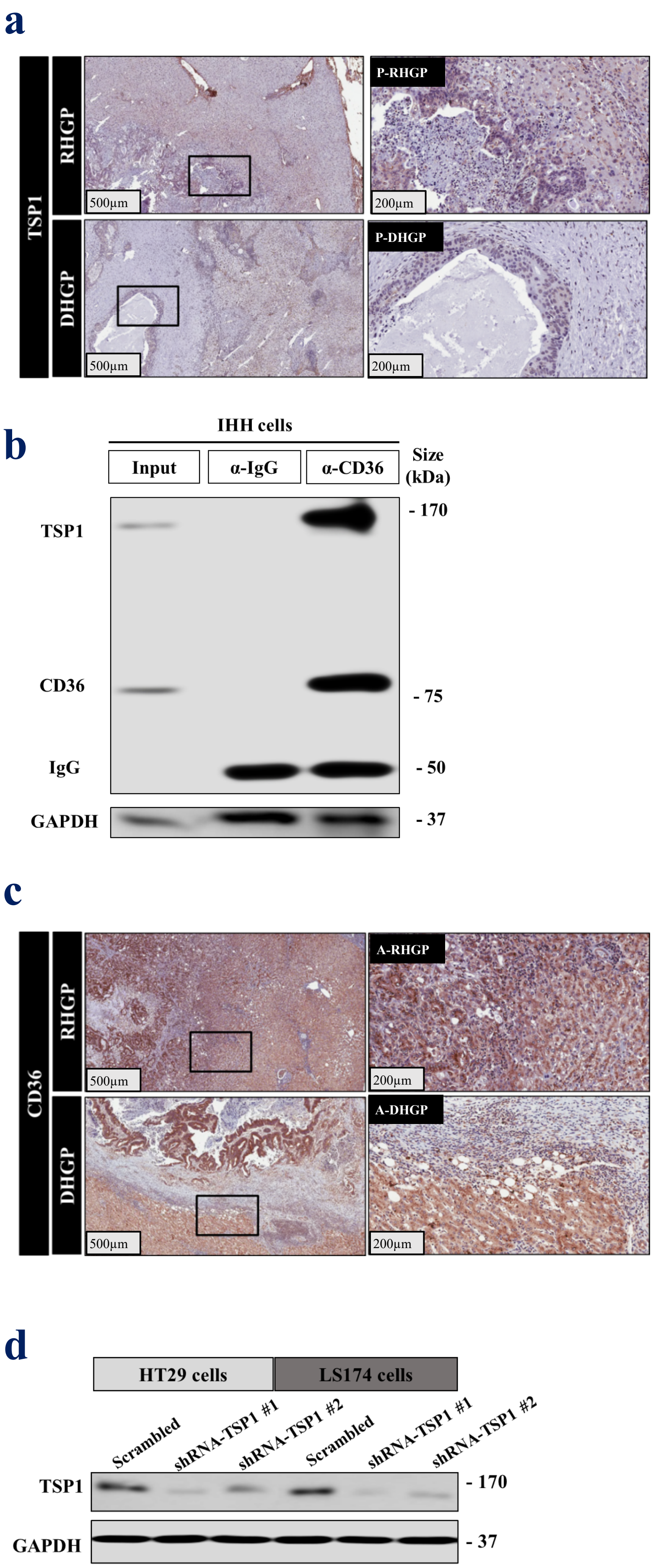

Rada *et al.*, Supplementary Figure 4

Supplementary Figure 4. TSP1 and its receptor (CD36) overexpressed in the replacement CRCLM lesions. a. Representative immunohistochemistry images of TSP1 in CRCLM (chemo+bev) lesions (left panel). Right panel shows the positivity [total number of positive pixels/total number of pixels] was measured using an optimized Aperio algorithm (mean + SD). P-RHGP=Peripheral tumour cells in RHGP lesions, P-DHGP=Peripheral tumour cells in DHGP lesions. **b.** Western blotting shows co-immunoprecipitation of CD36 and TSP1 using either anti-IgG or anti-CD36 antibody compared. **c.** Immunohistochemistry staining of chemonaïve CRCLM lesions with CD36 antibody. Right panel shows the positivity [total number of positive pixels/total number of pixels] was measured using an optimized Aperio algorithm (mean + SD). A-RHGP=Adjacent hepatocytes to tumour lesion in RHGP, D-RHGP= Distal hepatocytes to tumour lesion in RHGP, A-DHGP=Adjacent hepatocytes to tumour lesion in DHGP, D-DHGP= Distal hepatocytes to tumour lesion in DHGP. **d.** Immunoblotting represents colorectal cancer (HT29 and LS174) cells expressing either scrambled and TSP1 shRNA.

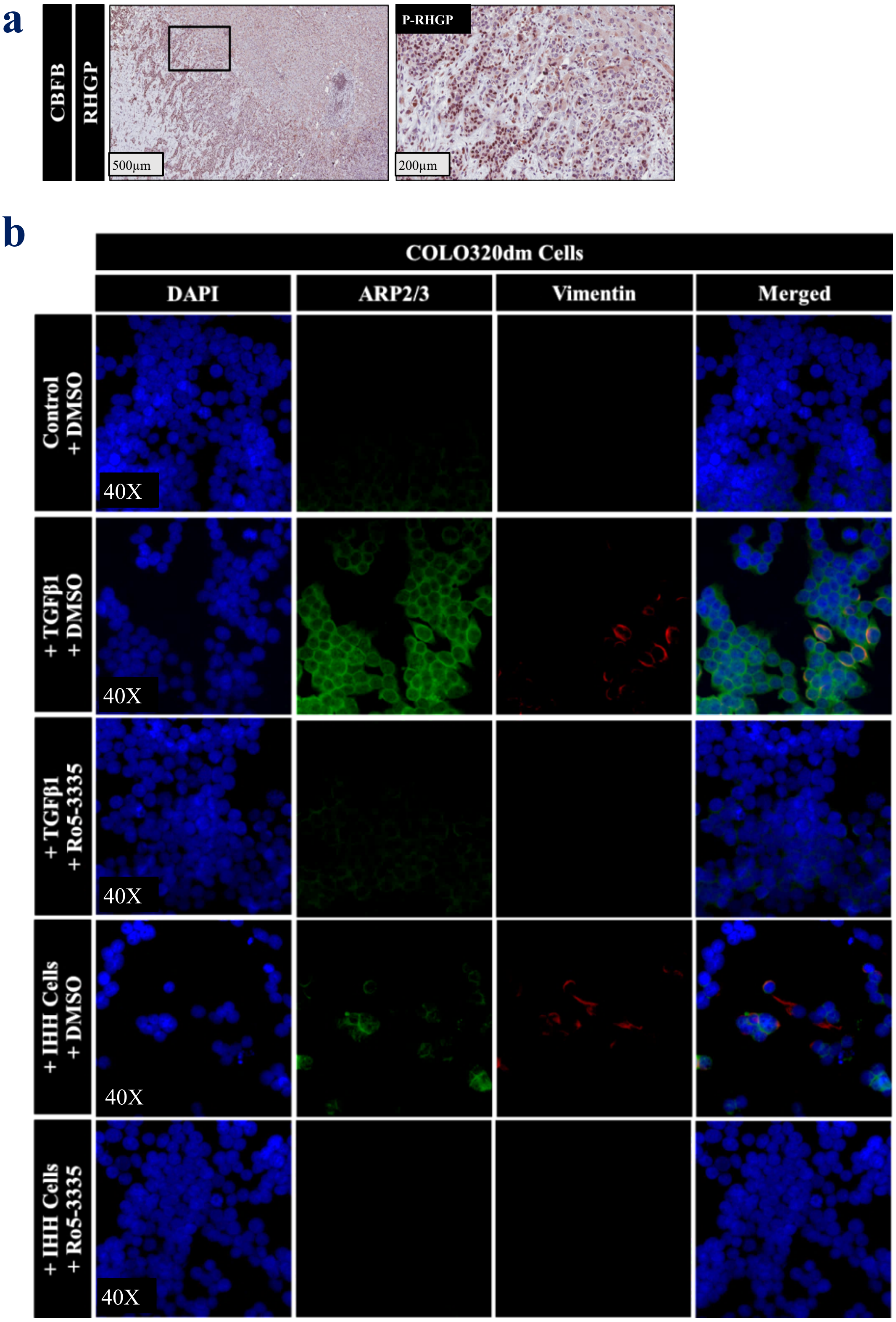

Rada *et al*., Supplementary Figure 5

Supplementary Figure 5. RUNX1 inhibition diminishes TGFβ1-dependent upregulation of ARP2/3 and vimentin in colorectal cancer cells. a. Immunohistochemistry staining of chemonaïve RHGP-CRCLM lesions staining CBFβ. **b.** Immunofluorescence staining of colorectal COLO320dm cancer cells showing the effect of RUNX1 inhibitor (Ro5-3335, 0.5µM) on ARP2/3 (green) and vimentin (red) expression in the presence of TGFβ1 (100pM) or co-cultured hepatocyte (IHH) cell line.

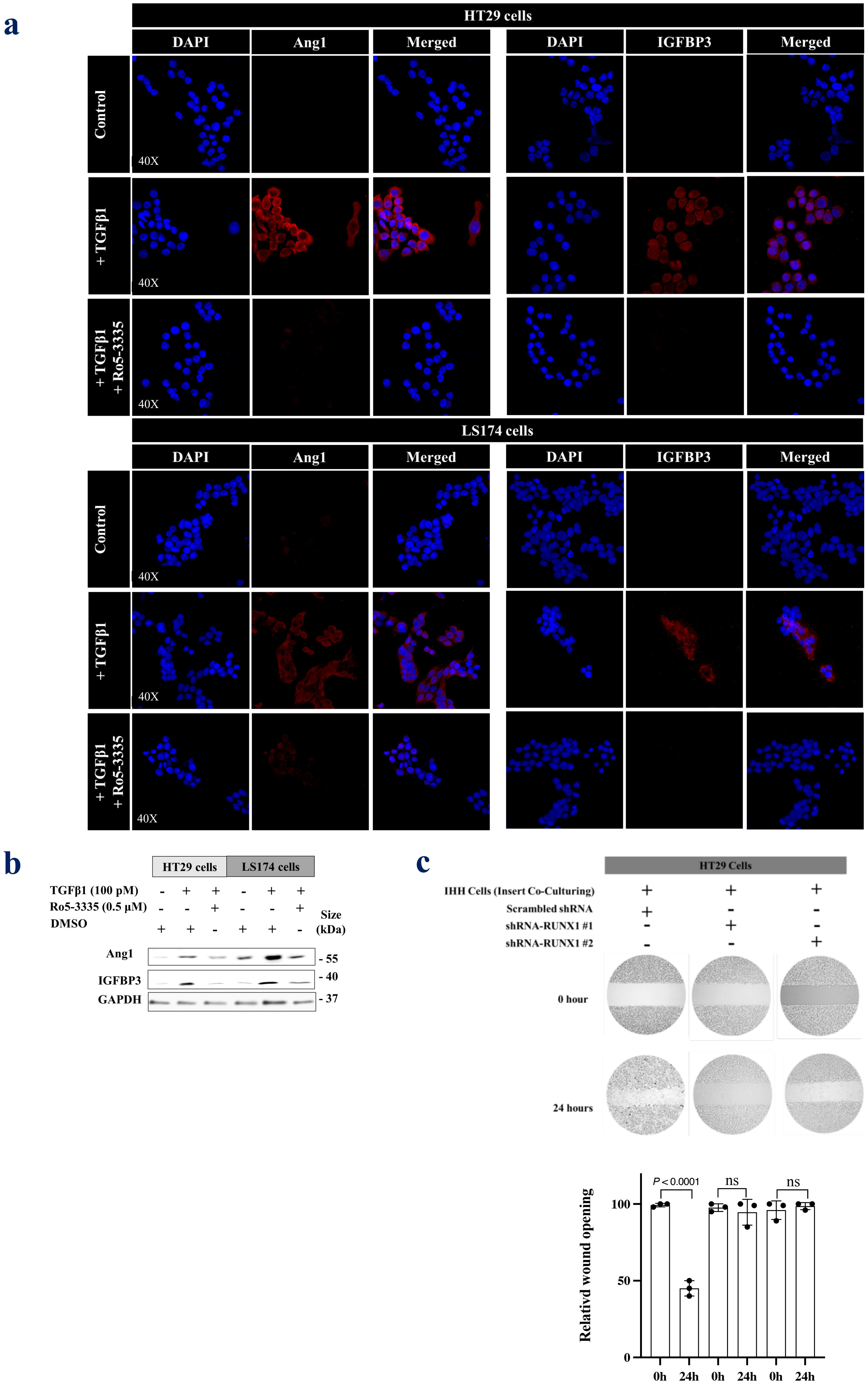

Rada *et al.*, Supplementary Figure 6

Supplementary Figure 6. Exposing cancer cells to TGFβ1 promotes RUNX1-dependent expression of antiangiogenic molecules, as well as motility. a. Immunofluorescence staining and immunoblotting (**b.**) of colorectal HT29 and LS174 cancer cells showing the effect of RUNX1 inhibitor (Ro5-3335, 0.5μM) on TGFβ1-dependent expression of anti-angiogenic molecules including Ang1 and IGFBP. **c.** HT29 cancer cells expressing either scrambled or RUNX1 shRNA grown to confluence, followed by scratch with pipet tips and subsequent incubation for 24 hours in the presence co-cultured hepatocyte (IHH) cell line. Data are presented as the mean ± SD.

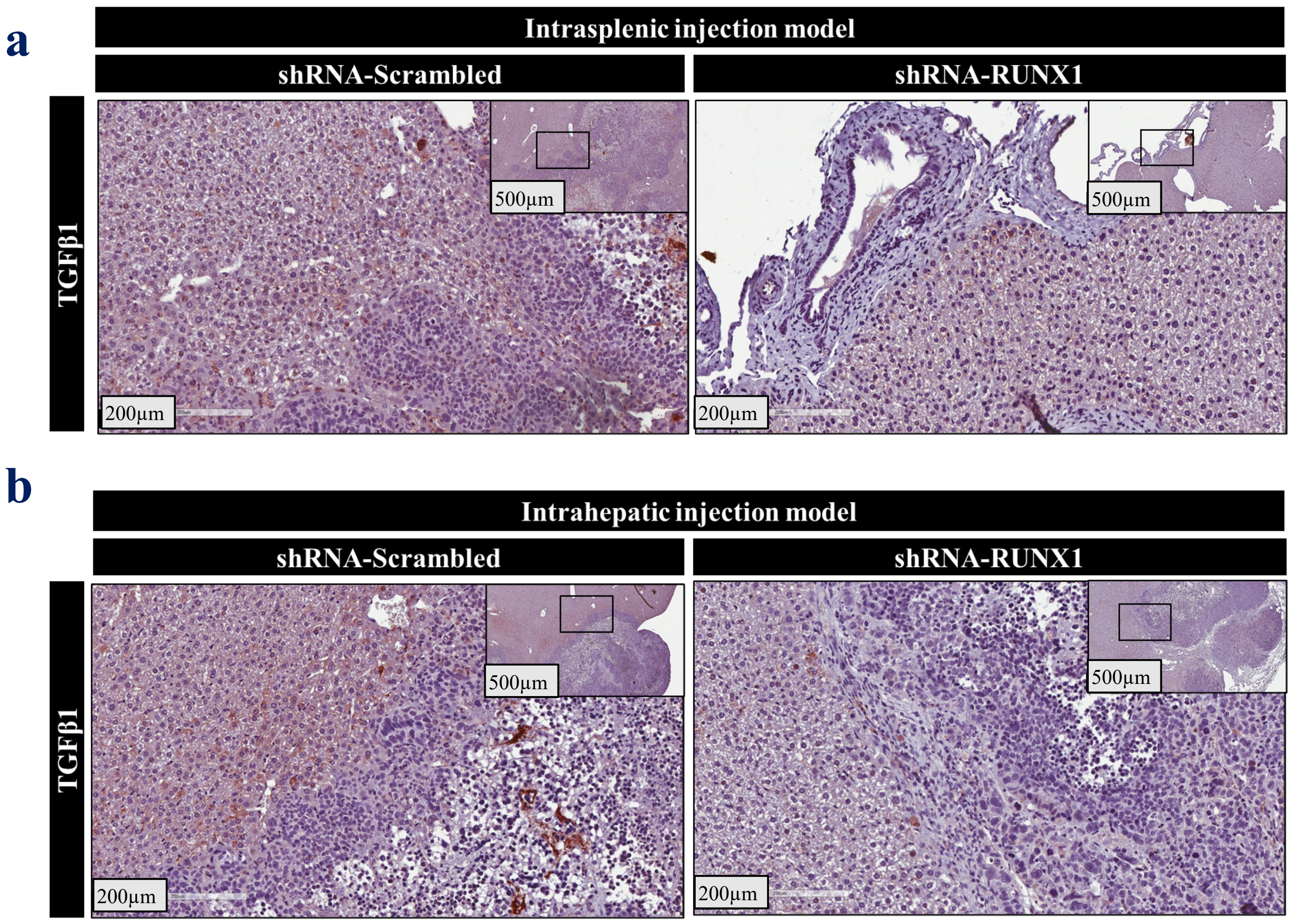

Rada *et al.*, Supplementary Figure 7

Supplementary Figure 7. The expression of TGFβ1 in mouse hepatic tumour lesions. Immunohistochemical staining showing TGFβ1 expression in hepatic tumour sections that generated from **(a.)** intrasplenically and (**b.)** intrahepatically injected mice with colorectal HT29 cancer cells.

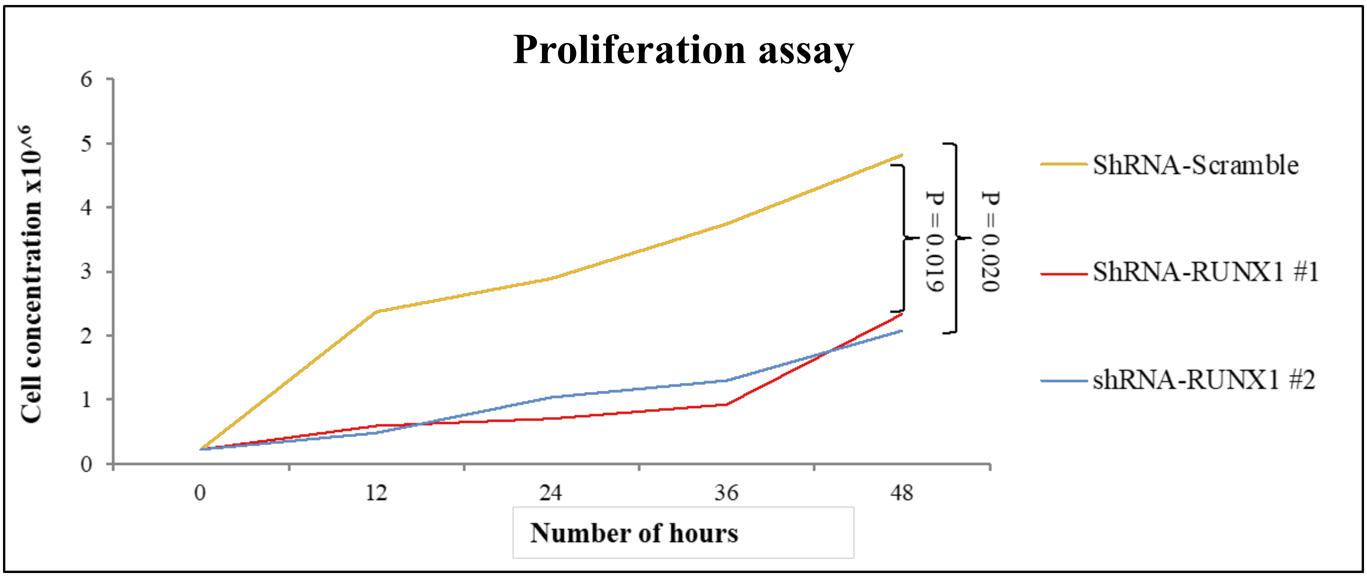

Rada *et al*., Supplementary Figure 8

Supplementary Figure 8. RUNX1 knockdown attenuated cell proliferation in colorectal cancer cell. Cell growth rates were measured for HT29 cancer cells expressing either shRNA-scrambled or shRNA-RUNX1 (#1 or #2) every 12 hours for 48 hours.

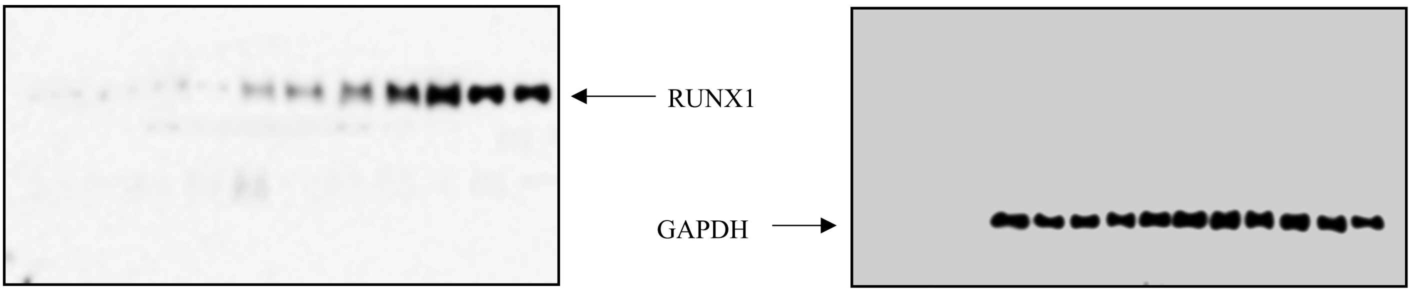

Rada *et al.*, Supplementary Figure 9 (Figure 1a)

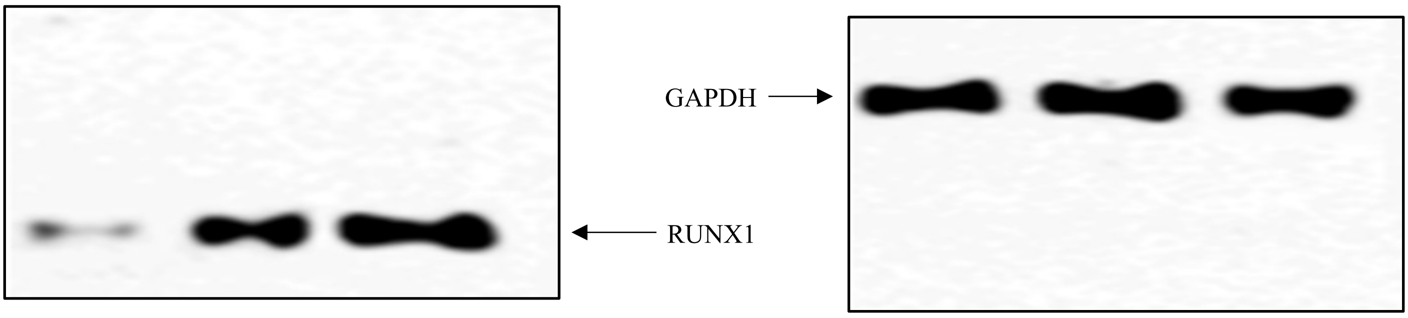

Rada *et al.*, Supplementary Figure 9 (Figure 2d)

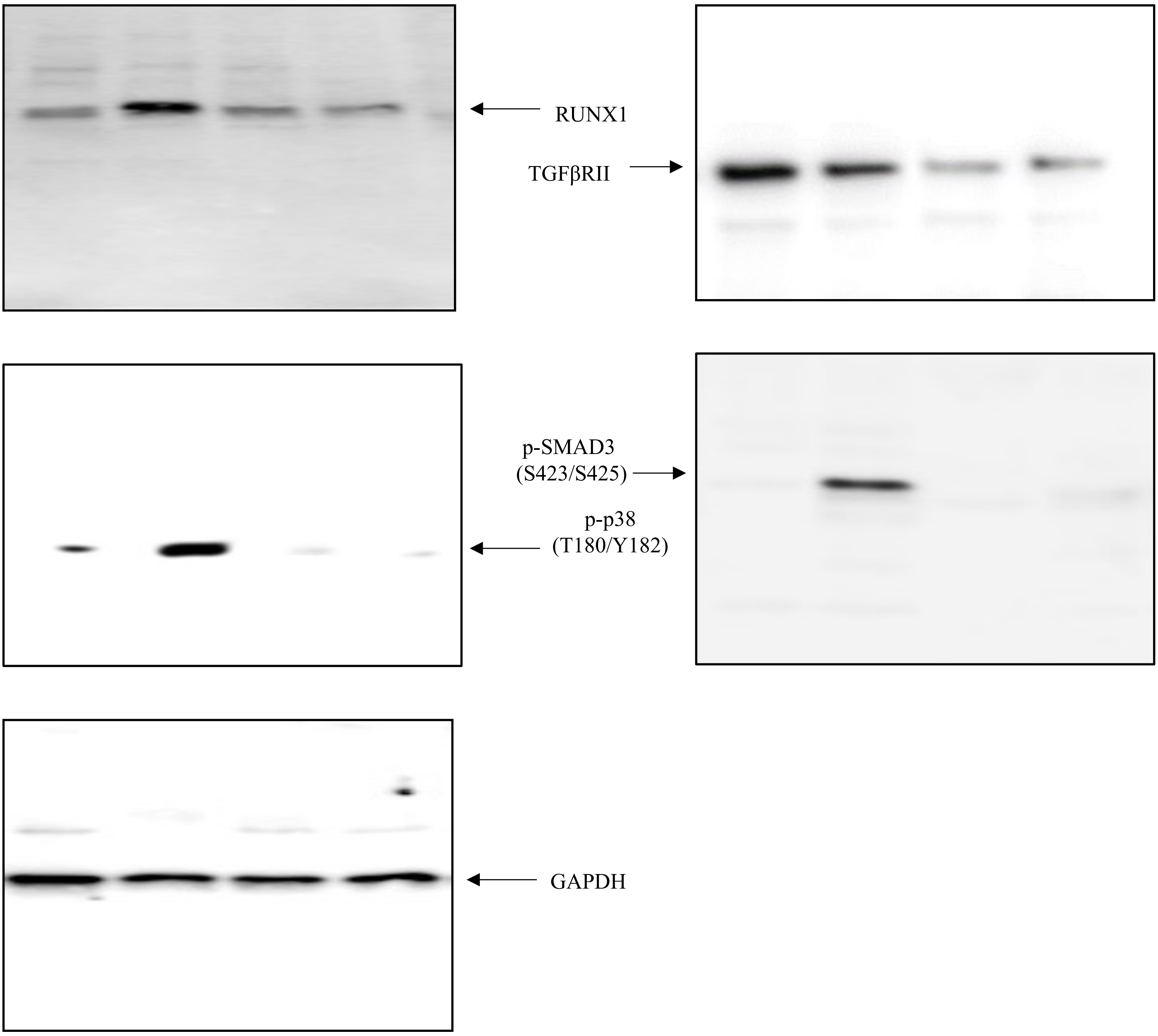

Rada *et al.*, Supplementary Figure 9 (Figure 2e)

Rada *et al.*, Supplementary Figure 9

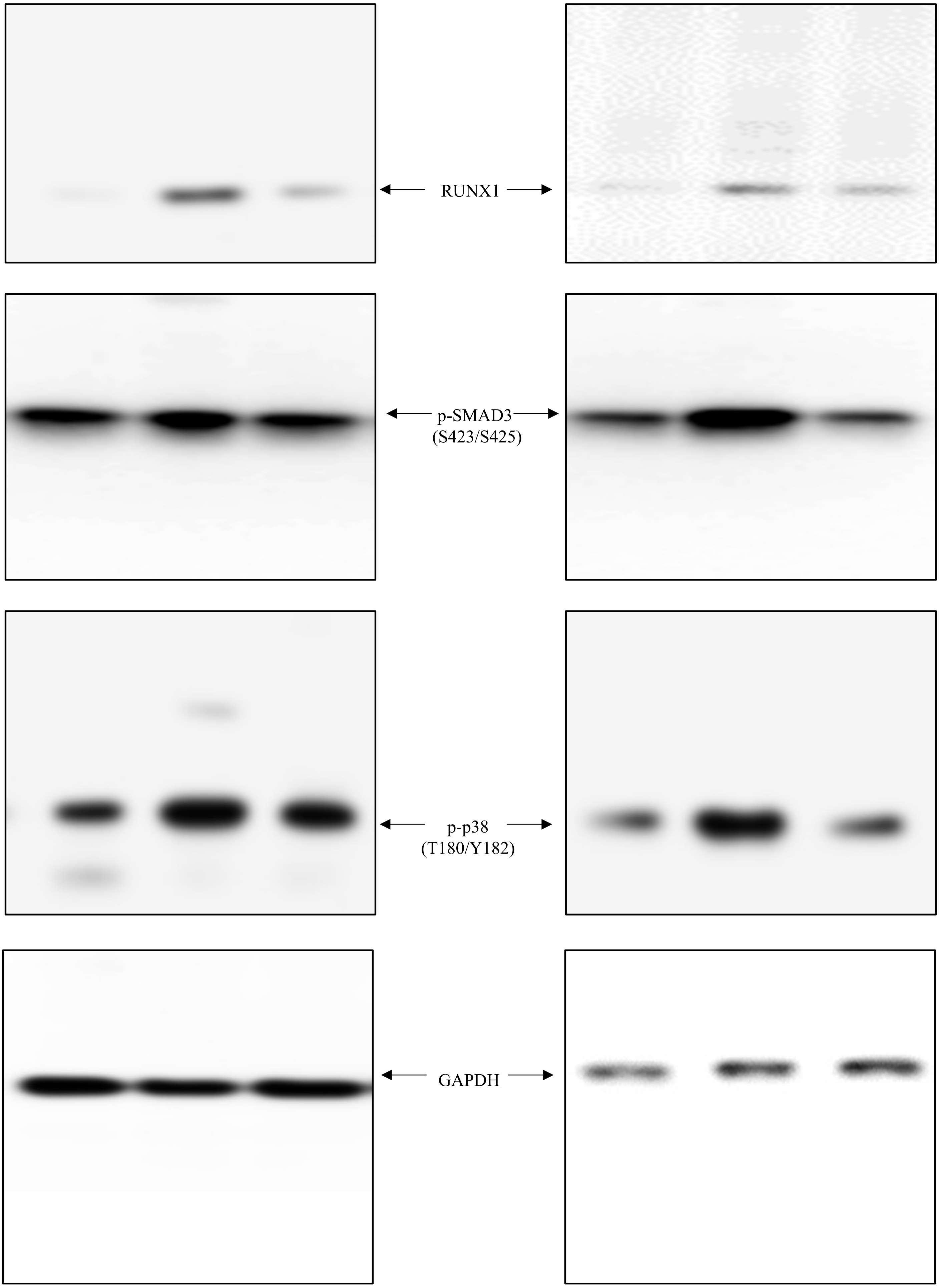

Rada *et al*., Supplementary Figure 9 (Figure 2f)

Rada *et al*., Supplementary Figure 9

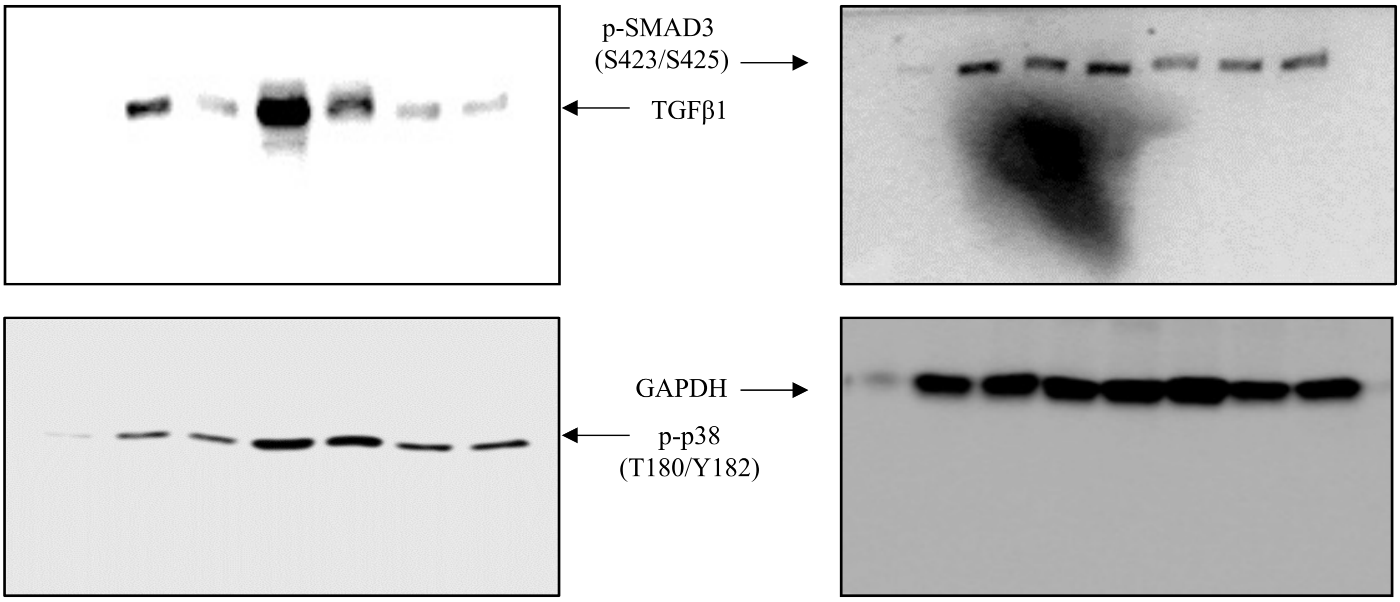

Rada *et al*., Supplementary Figure 9 (Figure 3b)

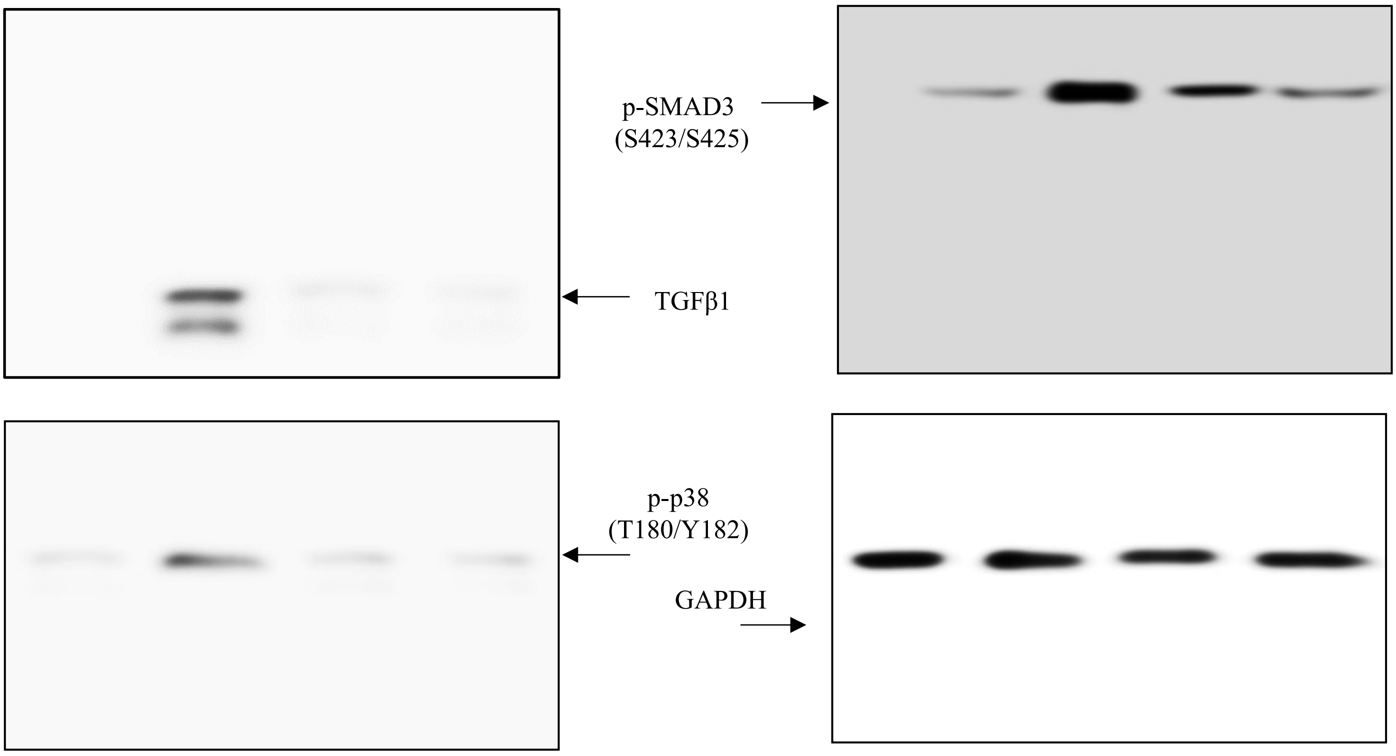

Rada *et al*., Supplementary Figure 9 (Figure 3d)

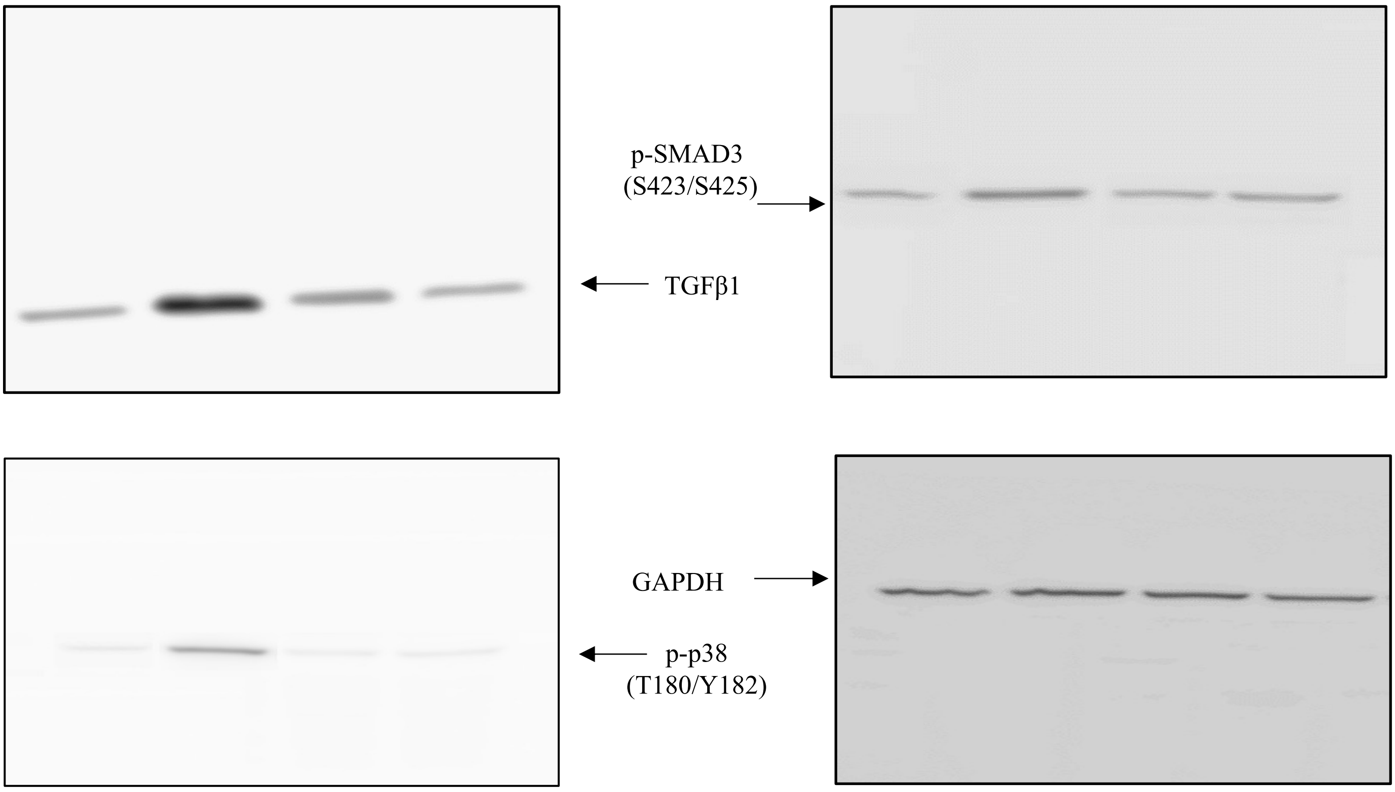

Rada *et al*., Supplementary Figure 9 (Figure 3e)

Rada *et al*., Supplementary Figure 9

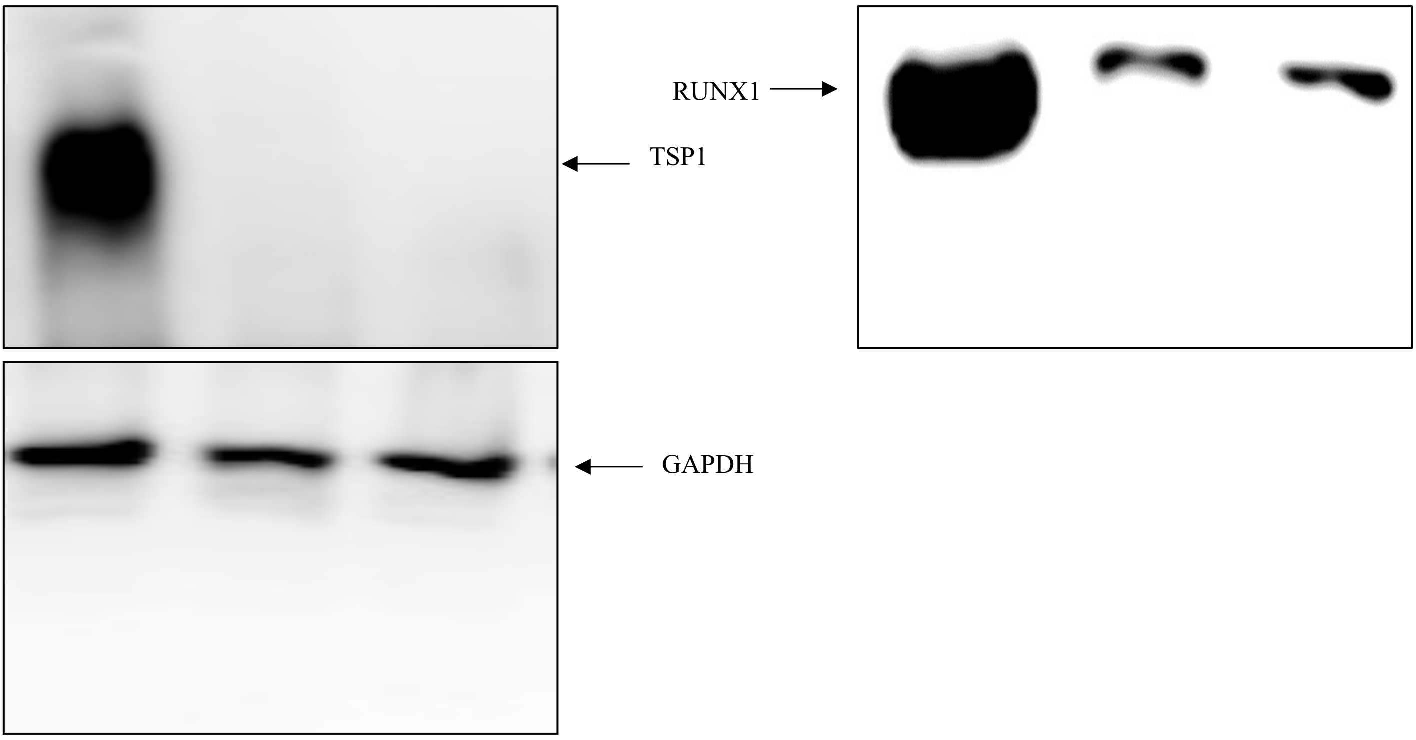

Rada *et al*., Supplementary Figure 9 (Figure 3f)

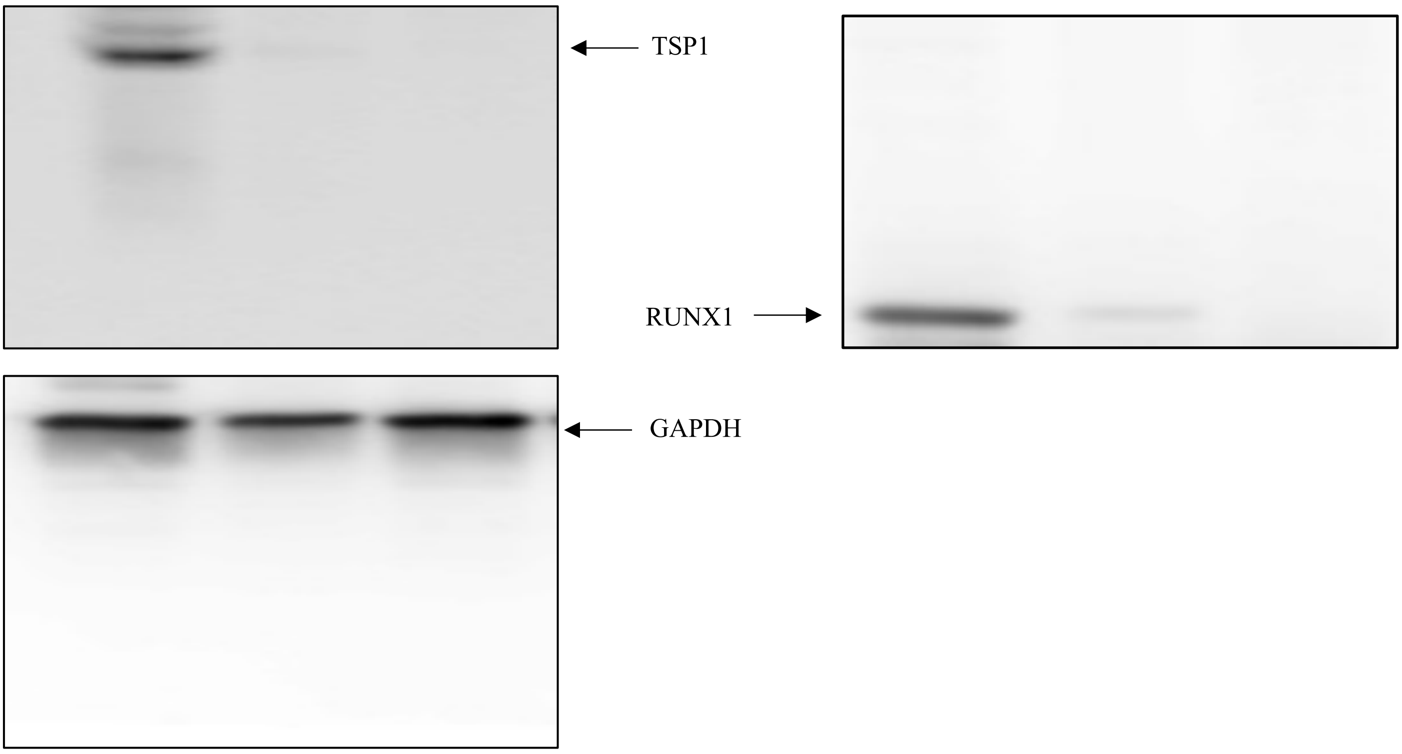

Rada *et al*., Supplementary Figure 9 (Figure 3g)

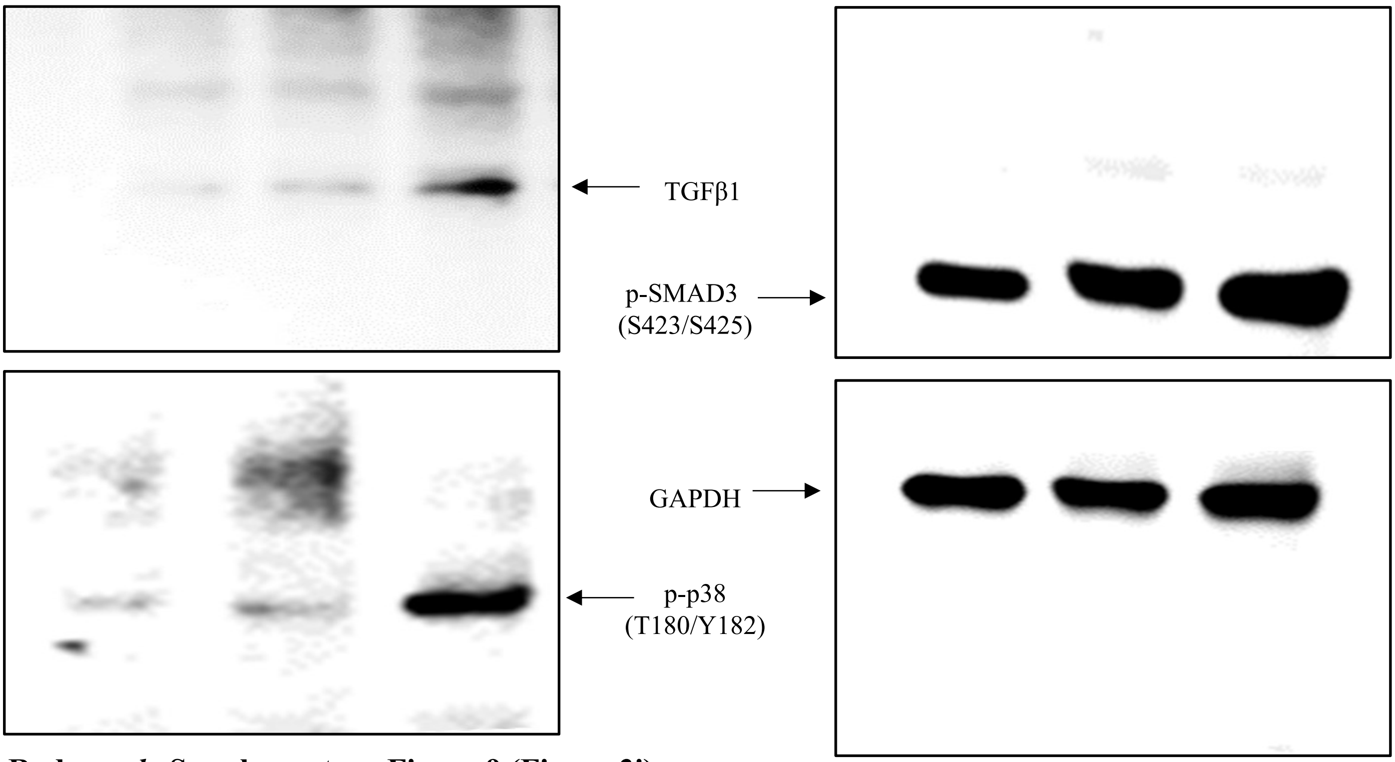

Rada *et al*., Supplementary Figure 9 (Figure 3i)

Rada *et al*., Supplementary Figure 9

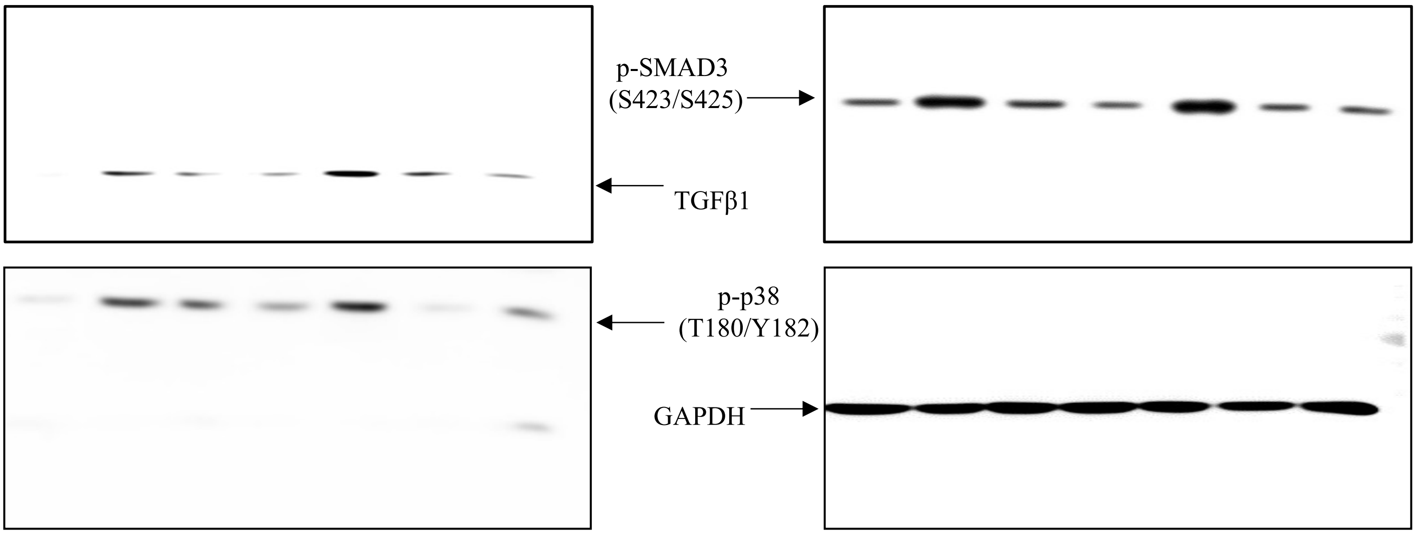

Rada *et al*., Supplementary Figure 9 (Figure 3j)

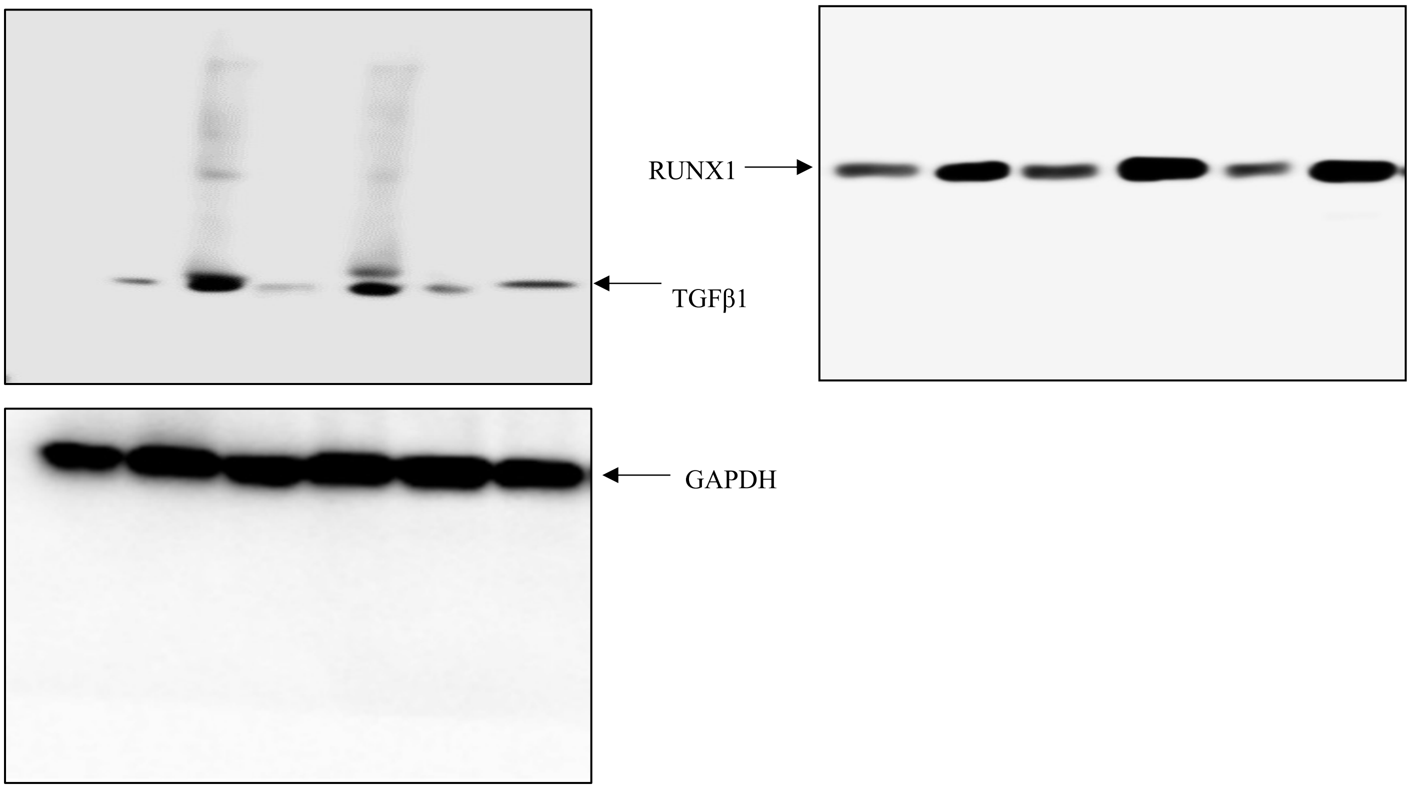

Rada *et al*., Supplementary Figure 9 (Figure 3k)

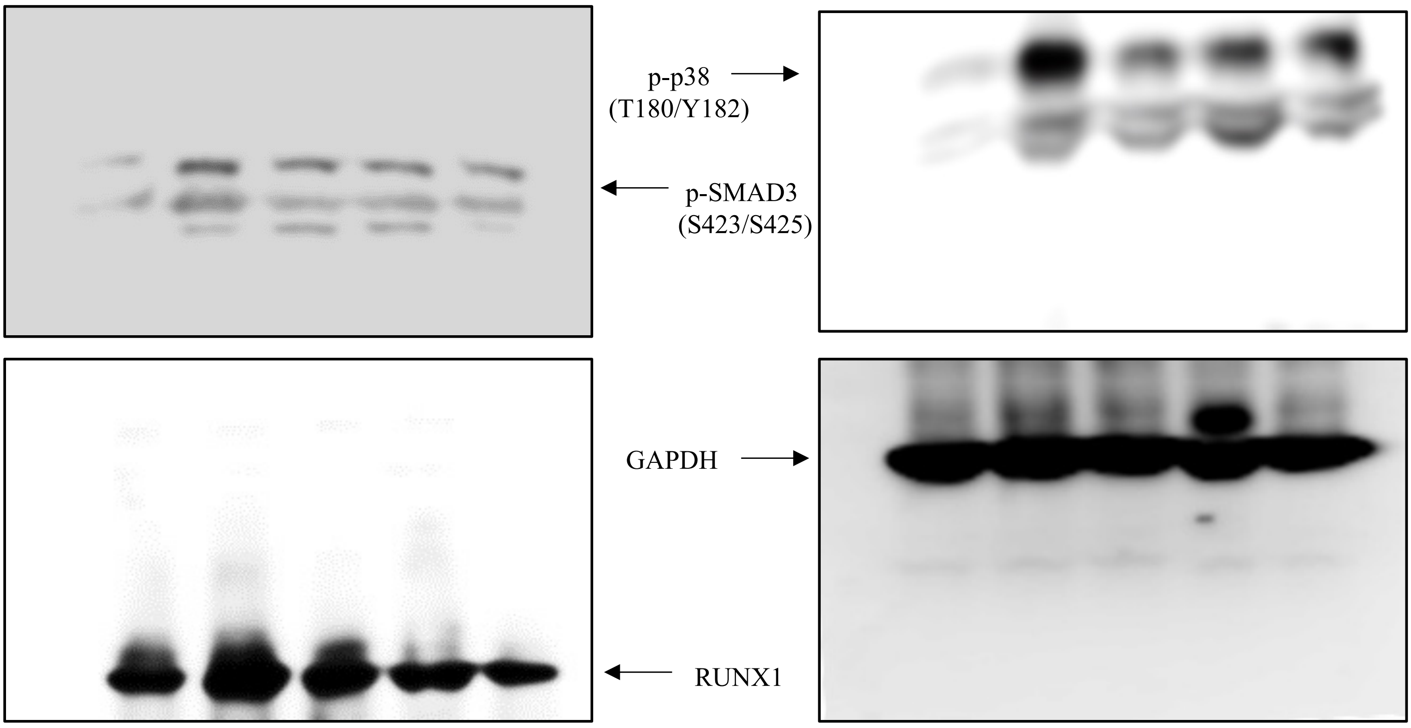

Rada *et al*., Supplementary Figure 9 (Figure 3l)

Rada *et al*., Supplementary Figure 9

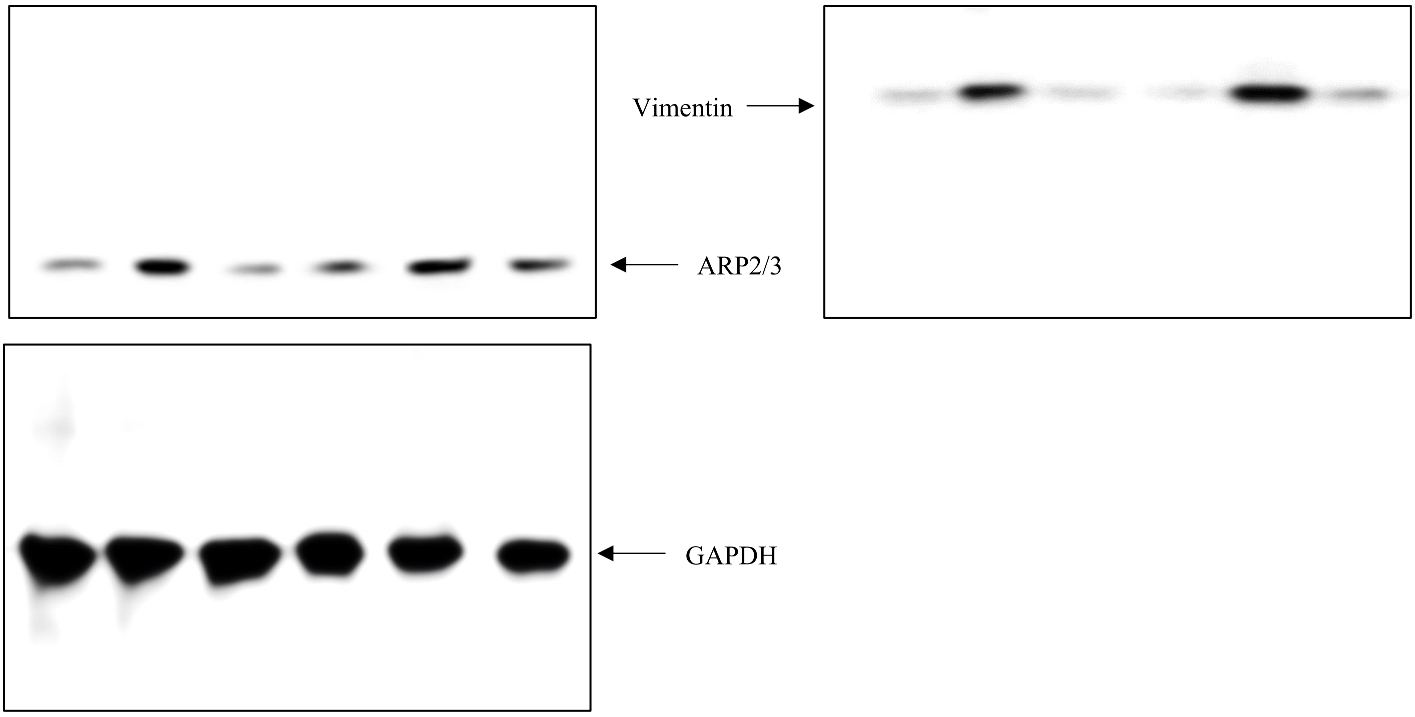

Rada *et al*., Supplementary Figure 9 (Figure 4a)

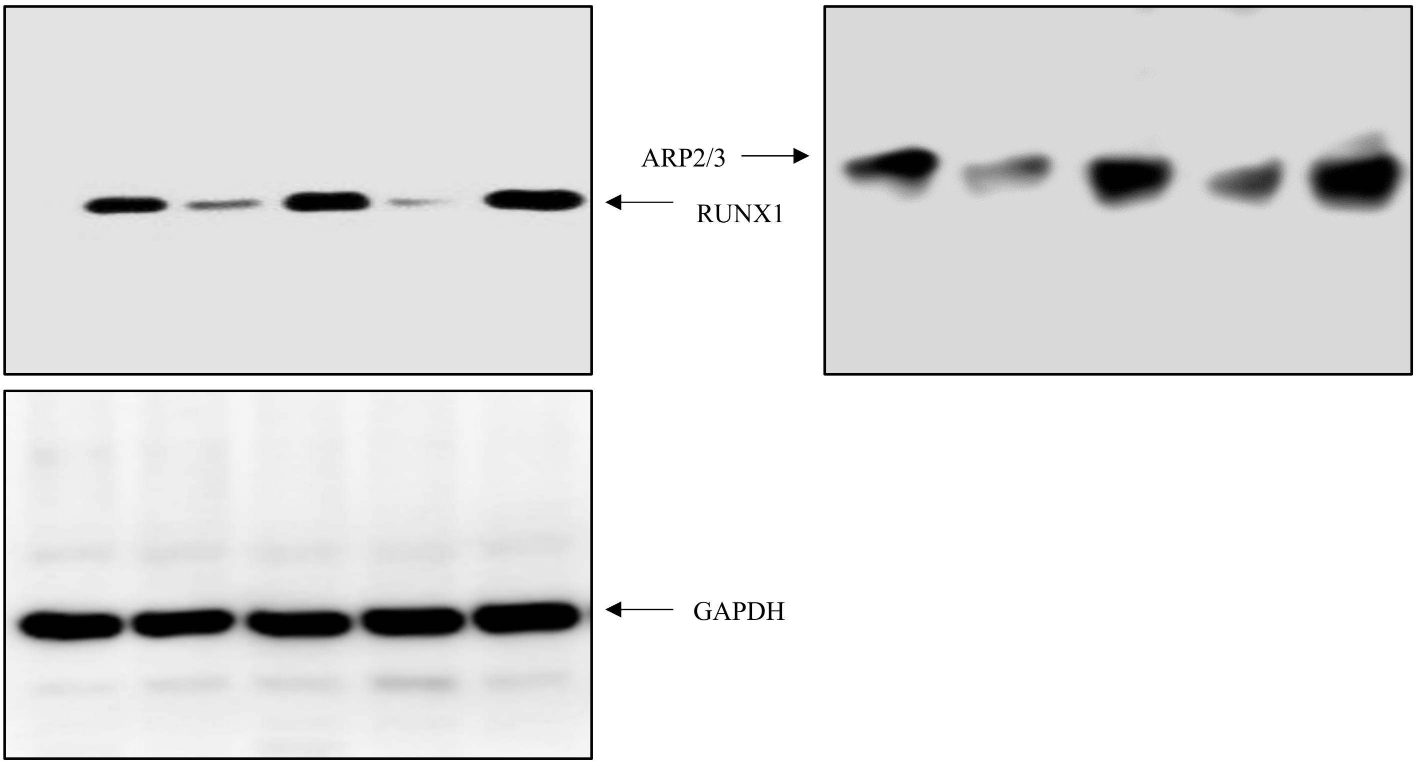

Rada *et al*., Supplementary Figure 9 (Figure 4e)

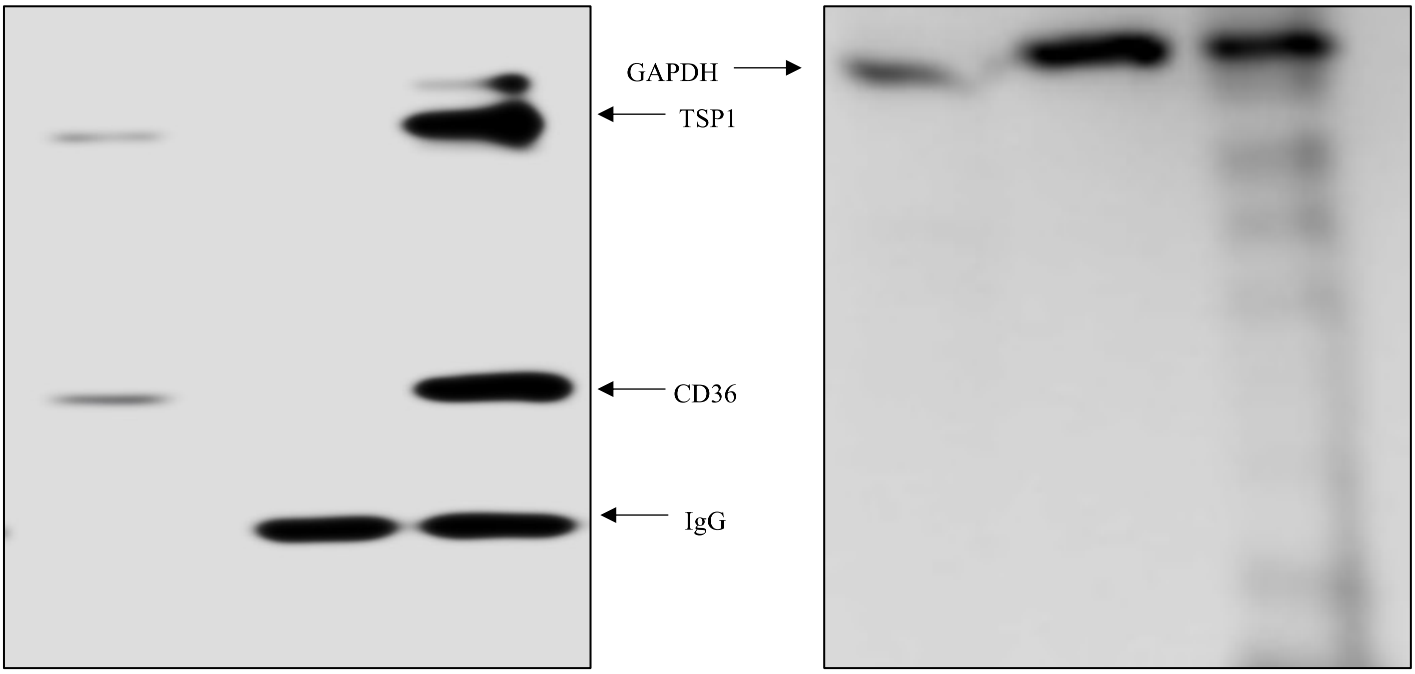

Rada *et al*., Supplementary Figure 9 (Supplementary Figure 4b)

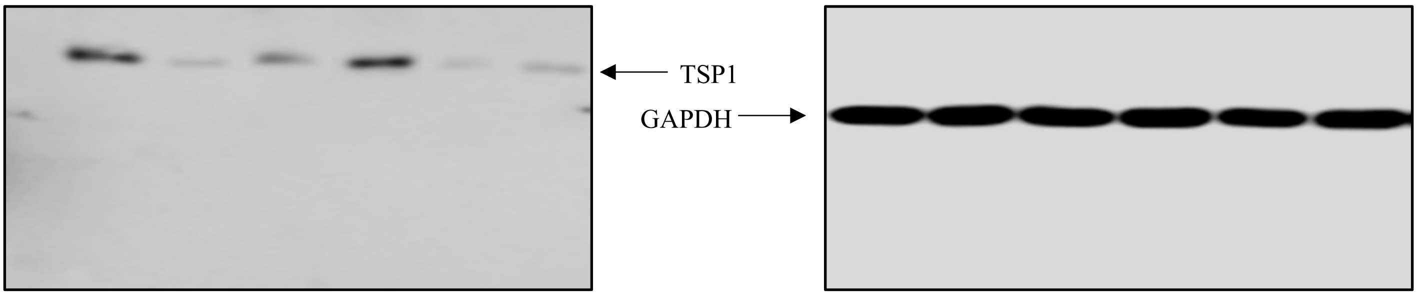

Rada *et al*., Supplementary Figure 9 (Supplementary Figure 4d)

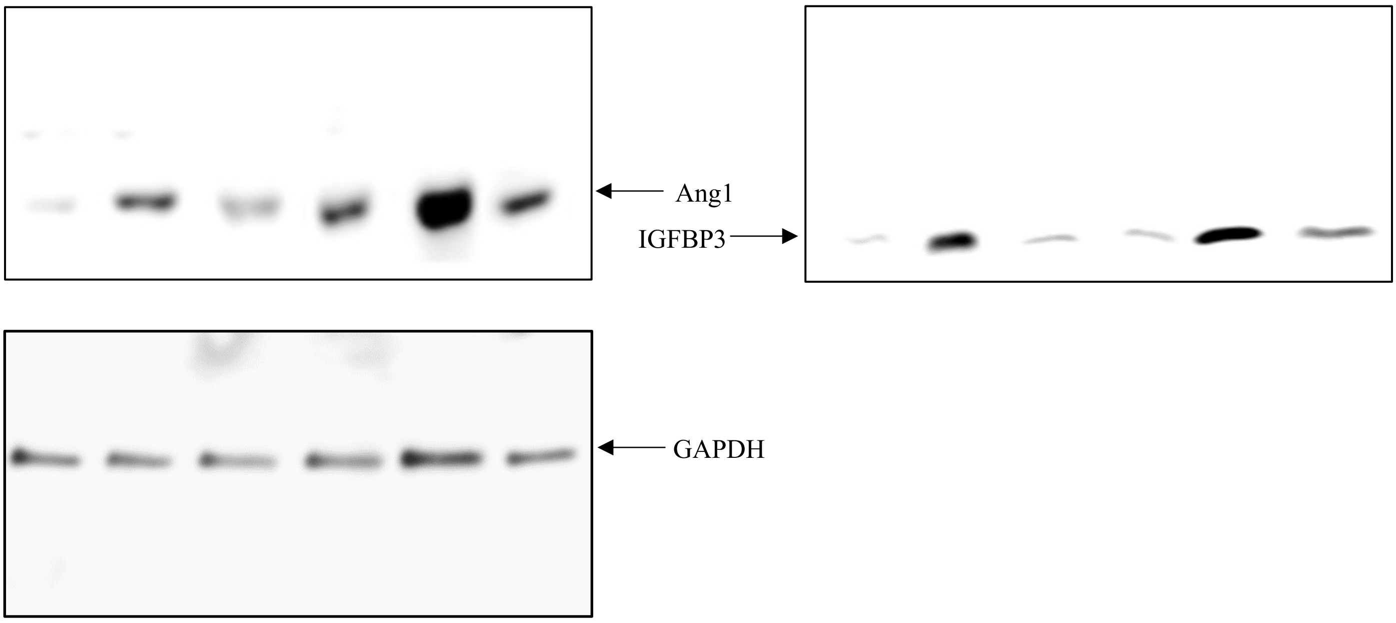

Rada *et al*., Supplementary Figure 9 (Supplementary Figure 6b)

Rada *et al*., Supplementary Figure 9
