# Supplementary material for: Runt related transcription factor-1 plays a central role in vessel co-option of colorectal cancer liver metastases
Source: Commun Biol. 2021 Aug 10;4:950. doi: 10.1038/s42003-021-02481-8 (PMC8355374; doi:10.1038/s42003-021-02481-8)
Supplement: Supplementary file 2 — Description of Supplementary Files [file 42003_2021_2481_MOESM2_ESM.pdf]

Description of Additional Supplementary Files

File name: Supplementary Data 1

Description: The data that shown in Figure 1C was collected from the publicly available data (GSE151165) that previously published by our lab (doi:10.1002/path.5449), as mentioned in the manuscript.
