# Supplementary material for: Runt related transcription factor-1 plays a central role in vessel co-option of colorectal cancer liver metastases
Source: Commun Biol. 2021 Aug 10;4:950. doi: 10.1038/s42003-021-02481-8 (PMC8355374; doi:10.1038/s42003-021-02481-8)
Supplement: Supplementary file 4 — Reporting Summary [file 42003_2021_2481_MOESM4_ESM.pdf]

nature research

Corresponding author(s): Dr Peter Metrakos

Last updated by author(s): May 6, 2021

Reporting Summary

Nature Research wishes to improve the reproducibility of the work that we publish. This form provides structure for consistency and transparency in reporting. For further information on Nature Research policies, see our Editorial Policies and the Editorial Policy Checklist.

Statistics

For all statistical analyses, confirm that the following items are present in the figure legend, table legend, main text, or Methods section.

n/a	Confirmed	
☐	☒	The exact sample size (*n*) for each experimental group/condition, given as a discrete number and unit of measurement
☐	☒	A statement on whether measurements were taken from distinct samples or whether the same sample was measured repeatedly
☐	☒	The statistical test(s) used AND whether they are one- or two-sided<br>*Only common tests should be described solely by name; describe more complex techniques in the Methods section.*
☒	☐	A description of all covariates tested
☒	☐	A description of any assumptions or corrections, such as tests of normality and adjustment for multiple comparisons
☒	☐	A full description of the statistical parameters including central tendency (e.g. means) or other basic estimates (e.g. regression coefficient) AND variation (e.g. standard deviation) or associated estimates of uncertainty (e.g. confidence intervals)
☒	☐	For null hypothesis testing, the test statistic (e.g. *F*, *t*, *r*) with confidence intervals, effect sizes, degrees of freedom and *P* value noted<br>*Give P values as exact values whenever suitable.*
☒	☐	For Bayesian analysis, information on the choice of priors and Markov chain Monte Carlo settings
☒	☐	For hierarchical and complex designs, identification of the appropriate level for tests and full reporting of outcomes
☒	☐	Estimates of effect sizes (e.g. Cohen's *d*, Pearson's *r*), indicating how they were calculated

Our web collection on statistics for biologists contains articles on many of the points above.

Software and code

Policy information about availability of computer code

Data collection	N/A
Data analysis	GraphPad Prism software version 7.0 (GraphPad Software, La Jolla, CA, USA) and Excel software for statistical analysis. To scan the IHC slides and score the staining positivity we used Aperio ImageScope V.11.2.0.780 software.

For manuscripts utilizing custom algorithms or software that are central to the research but not yet described in published literature, software must be made available to editors and reviewers. We strongly encourage code deposition in a community repository (e.g. GitHub). See the Nature Research guidelines for submitting code & software for further information.

Data

Policy information about availability of data

All manuscripts must include a data availability statement. This statement should provide the following information, where applicable:

- Accession codes, unique identifiers, or web links for publicly available datasets
- A list of figures that have associated raw data
- A description of any restrictions on data availability

The data that shown in Figure 1C was collected from publicly available data (GSE151165) that previously published by our lab, as mentioned in the manuscript

Field-specific reporting

Life sciences study design

All studies must disclose on these points even when the disclosure is negative.

Sample size	The sample size used in a study is determined based on the cost, time, convenience and need for it to offer sufficient statistical power. We believe that the number of samples that used in this study keep the chance of error at an acceptably low level and avoid making the experiments unnecessarily large. For all experiments involving human specimens, we have used the maximum number of samples we have able to access
Data exclusions	No data were excluded from the analyses
Replication	To verify reproducibility, we used different experiments and protocols
Randomization	Allocation was not random. The CRCLM specimens were allocated into two different groups including the replacement and desmoplastic histological growth patterns after scoring by histopathologist following the published consensus guidelines for scoring the histological growth patterns in CRCLM
Blinding	In majority of the experiments, the investigators were aware about the type of the CRCLM specimens that used for this study during analysis because the used patient specimens were previously scored for histological growth patterns by histopathologist after resection.

Reporting for specific materials, systems and methods

We require information from authors about some types of materials, experimental systems and methods used in many studies. Here, indicate whether each material, system or method listed is relevant to your study. If you are not sure if a list item applies to your research, read the appropriate section before selecting a response.

Materials & experimental systems

n/a	Involved in the study	
☐	☒	Antibodies
☐	☒	Eukaryotic cell lines
☒	☐	Palaeontology and archaeology
☐	☒	Animals and other organisms
☐	☒	Human research participants
☒	☐	Clinical data
☒	☐	Dual use research of concern

Methods

n/a	Involved in the study	
☒	☐	ChIP-seq
☒	☐	Flow cytometry
☒	☐	MRI-based neuroimaging

Antibodies

Antibodies used	TGFβ1 (Abcam, # ab27969; #ab215715), RUNX1 (LS Bio, # LS-C353932), E-Cadherin (R&D systems, #MAB1838-100), TGFBRII (Thermo Fisher, #PA5-35076), CD36 (abcam, #ab133625), TSP1 (abcam, #ab1823), CBFβ (LSBio, # LS-C342588), ARP2/3 (Millipore, #MABT95; Bioss, #bs-12524R), Ang1 (Abcam, #ab215715), Phospho-p38 (Thr180/Tyr182) (Cell Signalling Technology, #4631), Vimentin (abcam, ab16700), Phospho-Smad2 (Ser465/467) (Cell Signalling Technology, #3101), Cytokeratin 20 (abcam, #ab76126), IGFBP3 (Proteintech, #10189-2-AP), HSA (Santa Cruz, #SC5893), GAPDH (Abcam, # ab9485), TGFβ1 (Santa Cruz, # sc-130348), Phosphorylated SMAD3 (Ser423/425) (abcam, #ab52903).
Validation	We validated each antibody for IHC and IF in at least on type of tissues that suggested on the manufacturer's datasheet, and some of the used antibodies have been widely our lab over the last years.

Eukaryotic cell lines

Policy information about cell lines

Cell line source(s)	IHH cells were a generous gift from Dr Nabil G. Seidah at Montreal Clinical Research Institute (IRCM). Human colorectal cancer (HT29, LS174, LS180, SW620, COLO320dm) cell lines were a gift kindly supplied by Dr Alex Alex Gregorieff (Cancer Research Program, McGill University). HCT116 and HEK293T packaging cells were kindly provided by Dr Daniela Quail and Dr Peter Siegel respectively (Rosalind and Morris Goodman Cancer Research Centre, McGill University).
Authentication	non of the cell lines used were authenticated
Mycoplasma contamination	The used cell lines were tested negative for mycoplasma (MycoAlert Mycoplasma Detection Kit; Lonza)
Commonly misidentified lines (See ICLAC register)	N/A

Animals and other organisms

Policy information about studies involving animals; ARRIVE guidelines recommended for reporting animal research

Laboratory animals	We used 4- to 6-week old SCID Beige female mice for our experiments
Wild animals	This study did not involve wild animal
Field-collected samples	This study did not involve samples collected from the field
Ethics oversight	All animal experiments were conducted under a McGill University approved Animal Use Protocol in accordance with guidelines established by the Canadian Council on Animal Care.

Note that full information on the approval of the study protocol must also be provided in the manuscript.

Human research participants

Policy information about studies involving human research participants

Population characteristics	We have used specimens from chemonaive as well as treated CRCLM patients with chemotherapy and anti-angiogenic agent (Bevacizumab). All samples were obtained from McGill University Health Centre (MUHC) Liver Disease Biobank
Recruitment	McGill University Health Centre (MUHC) Liver Disease Biobank has recruited the CRCLM patients and informed consent was obtained from all patients
Ethics oversight	McGill University Health Centre Institutional Review Board approved protocol SDR-11-066

Note that full information on the approval of the study protocol must also be provided in the manuscript.
